# Molecular Mechanisms of Strength and Toughness in Slide-Ring Polymer Networks: Insights from Coarse-Grained Molecular Dynamics Simulations

**DOI:** 10.1021/acs.macromol.5c03515

**Published:** 2026-04-03

**Authors:** Zihan Tang, Weikang Xian, Ying Li

**Affiliations:** Department of Mechanical Engineering, 5228 University of Wisconsin-Madison, Madison, Wisconsin 53706, United States

## Abstract

Slide-ring (SR) polymer networks consist of cyclic molecules threaded onto polymer axial chains as movable cross-links, giving rise to exceptional extensibility and toughness. Yet, their mechanical response and fracture mechanisms remain unclear, particularly regarding how ring number, chain length, and cross-link number act in concert. Here, we use coarse-grained molecular dynamics simulations to systematically probe how these parameters govern deformation and failure in SR networks. We show that ring sliding is central to their mechanical performance, such as ultimate strength and toughness. Increasing ring number reduces ultimate strength and toughness by shortening sliding distances, suppressing chain orientation, and concentrating stress at chain ends. In contrast, increasing chain length enhances toughness and strength by enlarging sliding distances, promoting chain alignment, delaying fracture, and introducing entanglements that stabilize craze-like structures. Increasing cross-link number strengthens and toughens the networks by improving connectivity and stress transfer; however, the maximum sliding distance is reached at smaller strains, leading to higher stresses at small deformations but earlier failure at large strains. Void analysis further reveals that lower ring number suppresses void formation, whereas higher cross-link density promotes more homogeneous deformation. These molecular insights clarify strength-toughness trade-offs and guide the design of next-generation SR materials.

## Graphical abstract

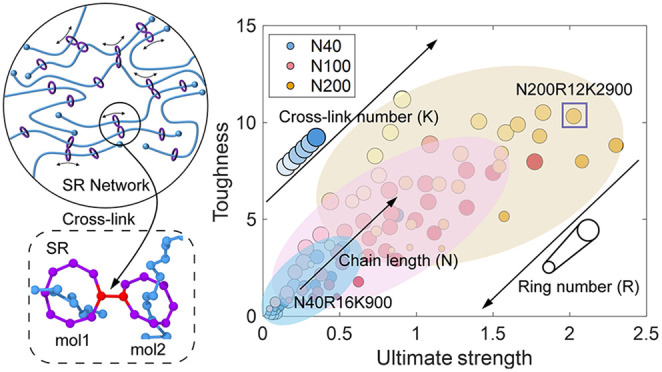

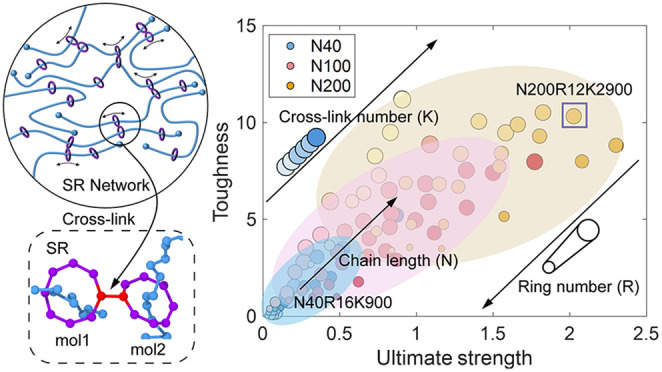

## 1 Introduction

Polymeric materials play indispensable roles in adhesives and coatings,^1,2^ biomedical systems,^3,4^ sustainable and recyclable materials,^5,6^ self-healing materials,^7,8^ soft robotics,^9^ and flexible electronics.^10–12^ Their widespread application critically depends on achieving desirable mechanical properties, particularly high strength and toughness.^13–16^ However, conventional fixed-cross-link (FC) polymer networks often suffer from an intrinsic trade-off: strong networks tend to be brittle with limited extensibility, whereas highly extensible networks usually sacrifice mechanical strength.^17–19^ This trade-off between strength and toughness has posed a longstanding challenge for designing advanced polymeric materials.

A variety of strategies have been proposed to overcome this limitation, such as nanoparticle–polymer composites,^20^ double network (DN),^21^ reversible cross-link,^22–27^ and movable cross-link.^28–31^ Nanoparticle–polymer composites are materials where nanoscale particles are embedded within the polymer network, creating a hierarchical structure with abundant interfaces.^20^ They improve mechanical properties mainly through stress transfer and redistribution, particle rearrangement, chain orientation, and energy dissipation.^32–34^ DN hydrogels, pioneered by Gong et al.,^21^ utilize a brittle network to dissipate energy while a ductile network sustains integrity,^21,35,36^ achieving high strength (MPa) and toughness (kJ/m^2^).^21^ Dynamic or reversible cross-links, which permit temporary bond breakage and reformation, can enhance energy dissipation and thus delay failure, also enabling self-healing and recyclability.^22–27^ More recently, movable cross-linking has emerged as a unique approach.^28–31^ These movable cross-links can enable topological rearrange of the network in response to deformation. In conventional FC networks, randomly positioned cross-links divide the constituent polymer chains into segments of uneven length, and the structural constraints induce heterogeneity and stress concentration.^37,38^ However, the movable cross-links allow stress redistribution along the chain, reducing local stress concentrations and enabling large deformations without premature fracture.^39,40^ As a result, polymer networks with movable cross-links exhibit strikingly enhanced extensibility and toughness compared to the FC networks, while maintaining comparable strength.

For example, Okumura and Ito fabricated slide-ring (SR) gels, in which cyclic molecules, typically α-cyclodextrins (α-CDs), are threaded onto long linear polyrotaxane chains, with bulky groups capping the chain-ends that prevent the rings from detaching.^41,42^ A network is formed via connecting pairs of rings into figure-eight cross-links. In our model, the axial chain length is defined by the number of beads per chain (*N*), as illustrated in Figure 1A­(i). The number of rings threaded onto each axial chain is denoted as *R* (Figure 1B), which determines the ring density *R*/*N*, defined as the percent ratio of covered length per axial chain. The number of cross-links between rings is represented by *K*, as shown in Figure 1C­(iv). During stretching, the ring molecules can slide along the axial chains, which enhances the structural homogeneity of the network and redistributes local stress.^39,40^ Thus, SR gels exhibit superior mechanical properties and hold great potential for broad applications in self-reinforcing and self-healing materials,^43^ and in tough, stretchable flexible electronic devices.^44,45^

**Figure 1:**
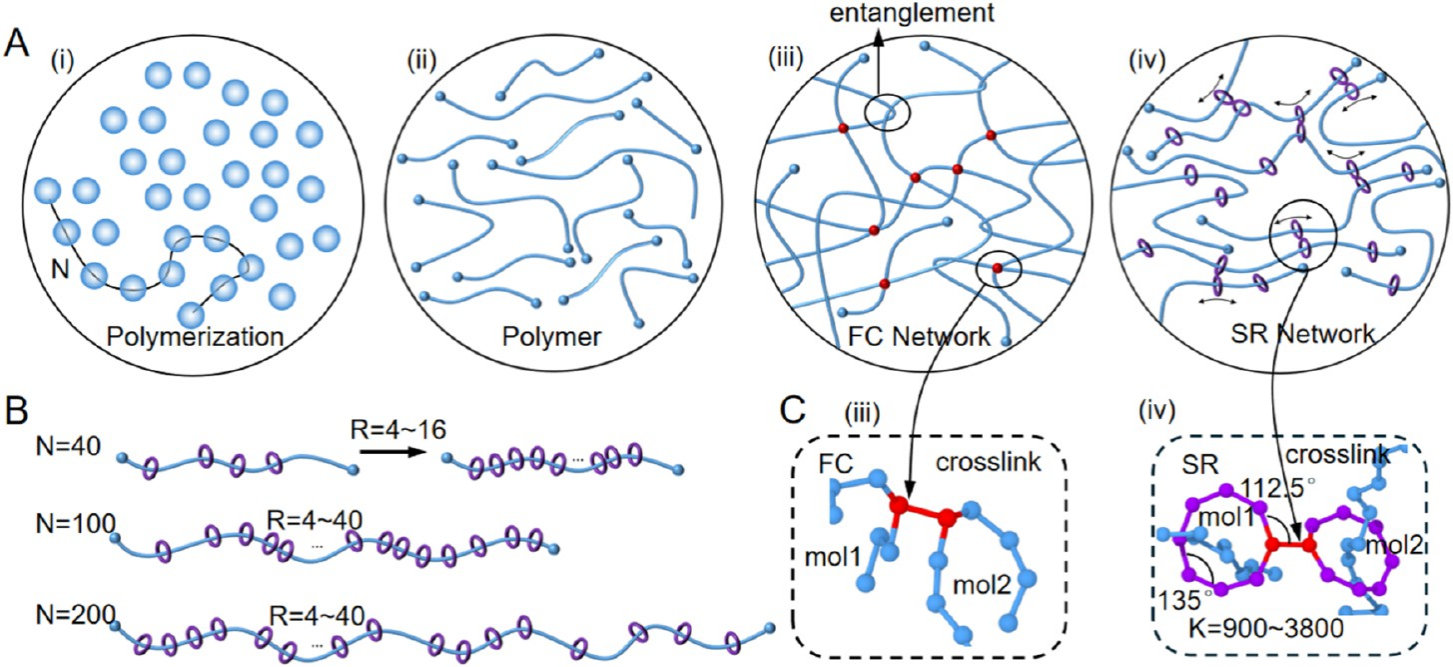
Simulation models of FC and SR polymer networks. (A) Simulation procedure: (i) chain generation by random-walk polymerization; (ii) linear polymers; (iii) formation of the FC network via intermolecular cross-links (red dots); (iv) formation of the SR network, where rings (purple) with a sufficiently large inner diameter can freely slide along the polymer’s axial chains and subsequently form cross-links. (B) SR models of different axial chain lengths (*N* = 40, 100, and 200) with varying numbers of rings per chain (*R* = 4–40). (C) Cross-linking for (iii) FC networks formed by direct bead–bead cross-linking between two chains (mol1 and mol2) and (iv) SR networks cross-linked between rings on different chains. The internal (135°) and external (112.5°) bond angles ensure that the rings remain rigid during deformation. To study the effect of cross-link number, the number of cross-links (*K*) is varied across 900, 1800, 2400, 2900, and 3800.

Despite these advances, the mechanical properties of SR networks are not yet fully understood. A key factor is the sliding distance of the rings, *N*_slide_, which strongly influences their mechanical response.^46–48^ The increase of *N*_slide_ enhances polymer network’s toughness in two major ways. First, ring density regulates the *N*_slide_. Jiang et al. and Kato et al. found that reducing ring density from ∼30% to below 5% allows rings to slide over more than two-thirds of the axial chain length.^49,50^ This resulted in a remarkable enhancement of mechanical performance, with networks sustaining elongations up to 1600% under stresses of ∼1 MPa while remaining fully recoverable.^49^ Other studies have also shown that decreasing ring density is consistently associated with improved toughness and extensibility in movable cross-link networks.^51–53^ Second, the axial chain length directly affects *N*_slide_. For example, increasing the molecular weight of the main chain from 35 to 100 kg/mol enlarges the sliding distance from approximately one-fifth to one-half of the chain length, which results in nearly a 5-fold increase in fracture energy.^46^ Other studies have similarly conlusions that longer chains substantially improve the mechanical performance of movable cross-link networks.^54–58^ However, the molecular-level mechanisms by which ring sliding affects fracture resistance of these polymer networks are still not fully understood.

To address this issue, molecular dynamics (MD) simulations provide a powerful approach to probe deformation and fracture processes of polymer networks at the molecular scale.^59^ MD simulations have been widely employed to investigate the ring dynamics,^60–65^ elasticity,^66–69^ fracture mechanism,^70^ recovery,^71^ and toughening mechanisms^72–74^ of SR networks. For example, Uehara et al. used coarse-grained MD (CGMD) simulations to examine the effect of *R* on fracture behavior.^70^ They reported that, during stretching, rings tend to slide and accumulate near the chain ends, leading to stress concentration and bond rupture. Reducing *R* were found to facilitate chain reorientation along the tensile direction, thereby enhancing toughness.^70^ However, these simulations did not explore the role of cross-link number between rings. Experimentally, cross-link density has been shown to significantly affect the mechanical performance of movable cross-link networks.^46,47,75–77^ For example, increasing the cross-link density reduces the fracture strain but enhances the ultimate strength, rendering the networks stiffer.^75^ Sawada et al. further found that higher cross-link density reduces the fracture energy.^76^ In contrast, Liu et al. found that fracture energy is independent of cross-link density and is instead governed by *N*_slide_.^46^ More recently, based on β-cyclodextrin trimers, the movable cross-link network exhibited toughness that first increased and then decreased with increasing cross-link density.^77^ These findings suggest that cross-link density has a pronounced influence on the mechanical response. Nevertheless, the molecular mechanisms underlying these effects have not yet been clarified. Gavrilov and Potemkin employed CGMD simulations to investigate the effect of varying *K* in SR networks.^66^ Their results showed rings on a polymer chain partition the chain into multiple segments, whose lengths depend on *K*. Increasing *K* makes both extremely short and extremely long distances more likely. This segmentation leads to stiffer network responses under larger *K*. However, the study was limited to small strains (smaller than 1.7), and the influence of *K* on fracture behavior has not yet been thoroughly explored.

In this study, we employed CGMD simulations to systematically investigate how ring number, axial chain length, and cross-link number determine the fracture behavior of SR networks. We unraveled the molecular mechanism of toughness in these unique polymer networks, and further correlated it with above design variables. We found that increasing *R* decreases both ultimate strength and toughness, whereas increasing *N* and *K* enhances these properties. We further quantified how ring sliding governs fracture behavior in SR networks and revealed the distinct roles of *R*, *N*, and *K* on deformation and failure mechanisms. Our findings not only provide new insights into the fracture mechanisms of SR networks at the molecular level, but also offer guiding principles to achieve high toughness and high strength of these polymer networks.

## 2 Model and Methods

The CGMD simulations were performed using the large-scale atomic/molecular massively parallel simulator (LAMMPS) package.^78^ All simulations were carried out under three-dimensional periodic boundary conditions, with box lengths denoted as *L*_*i*_ (*i* = *x*,*y*,*z*). The Lennard–Jones (LJ) reduced units were adopted, with length, energy, and mass defined as σ, *ε*, and *m*, respectively. The time unit was set as τ = σ­(*m*/ε)^1/2^. A standard bead–spring model was employed,^79^ consisting of 10^5^ beads at an initial density of 0.85/σ^3^. Polymer chains were generated by a random-walk growth method, starting from an initial bead and propagated by random placement of subsequent beads (Figure 1A­(i) and (ii)). The systems were equilibrated under the NVT ensemble for 5 × 10^6^τ at a reduced temperature of 1.0, using a Nosé-Hoover thermostat to control the temperature. The integration time step was set to 0.005τ. Subsequently, to accelerate equilibration, we employed the *fix bond/swap* algorithm in LAMMPS^80^ for 5 × 10^6^τ, which temporarily allows bonds to exchange between nearby beads using the Monte Carlo rules.^81,82^ This procedure effectively facilitates chain relaxation by enabling segmental rearrangements, thereby reducing the equilibration time. Once equilibrium was achieved (Figure S1), cross-links were introduced. For the FC networks (Figure 1A­(iii)), *R* beads along each linear chain were randomly selected as reactive beads. Cross-linking was carried out, where a new bond was formed if the distance between two reactive beads on different chains was less than 1.3σ.^67,69,83,84^ The simulation time of the cross-linking reaction was 3 × 10^6^τ. For the SR networks (Figure 1A­(iv)), *R* beads on each axial chain were attached to eight-bead ring molecules, whose inner diameter was sufficiently large to allow free sliding along the axial chains.^61,70,71^ The system was gradually relaxed by increasing the ring bond length from 0.6σ to 1.0σ. One in eight beads on each ring was assigned as a reactive bead. Intermolecular cross-links were subsequently formed between rings, yielding the SR networks (Figure S2).

Experimentally, chain length is commonly varied through changes in molecular weight.^46,55^ In our simulations it is controlled by *N* = 40, 100, and 200, with the corresponding number of chains (CN) set to 2500, 1000, and 500, respectively. Thus, three different chain lengths were chosen to study the effect of *N*. *R* was varied from 4 to 40 (Figure 1B) to investigate the effect of ring density. *K* was tuned by controlling the total number of newly formed bonds (*K* = 900, 1800, 2400, 2900, or 3800), as shown in Figure 1C. Each system is labeled as NRK. For example, N40R8K3800 refers to the system with *N* = 40, *R* = 8 and *K* = 3800. All the systems in this study were summarized in Table 1. After cross-linking, each system was further equilibrated for 5 × 10^5^ steps under NVT conditions and 5 × 10^5^ steps under NPT conditions, with the pressure set to zero. The temperature and pressure were controlled by the Nosé–Hoover thermostat and barostat, respectively. Additional details of the simulation protocol and the equilibration test are provided in the Supporting Information.

**Table 1:** Structural Parameters of the Simulated Polymer Networks

network	*N*	CN	*R*	*K*
SR	40	2500	4–16	900–3800
	100	1000	4–40	
	200	500	4–40	
	200	5000	12	29,000
FC	200	5000	12	29,000

The total potential energy of the system consisted of bonded and nonbonded contributions, defined as

1 $$E_{\mathrm{total}}=E_{\mathrm{bonded}}+E_{\mathrm{non‐ bonded}}=E_{\mathrm{bond}}+E_{\mathrm{angle}}+E_{\mathrm{LJ}}$$

where *E*_bonded_ includes the bond stretching term *E*_bond_ and the angular term *E*_angle_, which describe the connectivity of the axial chains and rings and the geometric constraints of the rings, respectively. The nonbonded contribution *E*_nonbonded_ is represented by a shifted LJ potential *E*_LJ_, accounting for excluded volume and weak intermolecular interactions. To allow for studies of bond breaking, the axial chain bonds were modeled using a quartic potential.^85–88,36^

2 $$E_{\mathrm{bonded}}\left(r_{\mathrm{ij}}\right)=K_{1}\left[{\left(r_{\mathrm{ij}}-r_{a}\right)}^{3}\left(r_{\mathrm{ij}}-r_{b}\right)\right]+U_{0}$$

where *r*_*ij*_ is the distance between the *i*th and *j*th bead, and the stiffness constant was set to *K*_1_ = 3600εσ^–4^. The parameters *r*_*a*_, *r*_*b*_ and *U*_0_ were 1.5σ, 2.5/3.0σ and 75.0ε, respectively.^85^ *r*_*a*_ is the cutoff distance beyond which the bond was removed, and *U*_0_ defines the energy barrier, as shown in Figure S5. *r*_*b*_ was selected such that the minimum of the quartic potential was located at 1.0σ, identical to the equilibrium bond length of the harmonic bonds used for ring molecules. These parameters enable the axial bonds to sustain finite extensibility while preventing unrealistic elongation. This makes the model well-suited for capturing bond-breaking events in polymer axial chains. For ring molecules, harmonic bonds with a force constant *K*_2_ of 500ε/σ^2^ and an equilibrium length of *r*_0_ = 1σ were employed.

3 $$E_{\mathrm{harmonic}}\left(r_{\mathrm{ij}}\right)=\frac{K_{2}}{2}{\left(r_{\mathrm{ij}}-r_{0}\right)}^{2}$$

To maintain ring rigidity, two angular constraints were used to preserve their geometry.^67,69,85^

4 $$E_{\mathrm{angle}}\left(\theta \right)=\frac{K_{3}}{2}{\left(\theta -\theta_{0}\right)}^{2}$$

where the force constant of *K*_3_ = 100.0εrad^–2^ with an equilibrium angle of 135° and 112.5° for two different types of angles, as shown in Figure 1C­(iv). These two constraints stabilize the internal and external bond angles, thereby ensuring that the rings remain rigid during deformation. Nonbonded interactions were described by a shifted LJ potential.^71,85^

5 $$E_{\mathrm{LJ}}\left(r_{\mathrm{ij}}\right)={4\varepsilon }_{\mathrm{ij}}\left[{\left(\frac{1}{r_{\mathrm{ij}}-\Delta r}\right)}^{12}-{\left(\frac{1}{r_{\mathrm{ij}}-\Delta r}\right)}^{6}-U\left(r_{cutoff}\right)\right]$$

All nonbonded interaction parameters were taken from Zhang et al’s work,^71^ as given in the Supporting Information. And these force field parameters will not allow ring–chain interpenetration or bond crossing, as shown in Figure S6.

To investigate deformation processes such as void formation and crazing, triaxial stress states were needed.^89–93^ Uniaxial extension was applied along the *x*-direction at a constant engineering strain rate of *L̇*_*x*_/*L*_*x*_ = 5 × 10^–5^/τ, while the *y*- and *z*-directions (*L*_*y*_ and *L*_*z*_) were maintained at constant lengths. Additional tensile tests along the *y*- and *z*-directions are provided in Figure S7, confirming the isotropic mechanical response. The applied strain was calculated as strain = *L*_*x*_/*L*_*x*0_ – 1. A Langevin thermostat with a damping coefficient of 2τ was applied to the *y*- and *z*-directions to maintain the target temperature 1.0 during the deformation process.^89,90^ The velocity-Verlet algorithm was employed to integrate the equations of motion. The nominal stress in the stretching direction was obtained from the virial stress tensor as^69,94^

6 $$\sigma_{N}=\sigma_{\mathrm{xx}}-\frac{1}{2}\left(\sigma_{\mathrm{yy}}+\sigma_{\mathrm{zz}}\right)$$

where σ_*xx*_ is the virial stress component along the loading direction, and σ_*yy*_ and σ_*zz*_ are the transverse stress components. While the local atomic-level stress was calculated from the per-atom virial stress σ_*i*_ normalized by the atomic volume *V*_*i*_, σ_local_ = σ_*i*_/*V*_*i*_. The atomic volume *V*_*i*_ was obtained using a Voronoi tessellation implemented in the LAMMPS command compute voronoi/atom.^95,95^ The per-atom virial stress was evaluated as^85^

7 $$\sigma_{i}=-\frac{1}{2}\sum_{j}\frac{dE_{\mathrm{total}}}{dx_{\mathrm{ij}}}x_{\mathrm{ij}}+\frac{1}{m}p_{\mathrm{ix}}p_{\mathrm{ix}}$$

where *x*_*ij*_ denotes the *x*-component of the vector connecting atom *i* to atom *j*. The term d*E*_total_/d*x*_*ij*_ represents the force component along the *x*-direction, while *p*_*ix*_ is the momentum of atom *i* along the same direction, and *m* is the bead mass. The first term, therefore, corresponds to the configurational contribution from interatomic forces, and the second term accounts for the kinetic contribution.

## 3 Results

### 3.1 Effect of Ring Number

To investigate the effect of *R*, the mechanical response of SR networks was compared. The chain length is *N* = 40 and the cross-link number is *K* = 3800. *R* was varied from 4 to 16, corresponding to ring density 10% to 40%, which is comparable to experimental systems where density typically ranges from 2% to 30%.^49,50^ The stress–strain curves (Figure 2A) reveal three distinct regimes. At strains below ∼3 for different *R* values, the stress monotonically increases without any bond rupture. Between strains of 3–6, bond breaking initiates, and the stress significantly rises until reaching its maximum value. Beyond this point, the SR networks progressively fail, and the stress drops to zero quickly. The bond-breaking profiles (Figure 2B) show that SR networks with fewer rings undergo delayed bond rupture, indicating that reducing ring density improves fracture resistance. The corresponding structural evolution (Figure 3A) illustrates how the SR network deforms. A representative ring trajectory (Figure 3B) highlights the sliding of rings along the axial chain and the eventual occurrence of bond rupture. Similar trends were also observed at different *N* and *K* (see Figures S8–S10), as given in previous simulations.^85^ The simulation results also agree well with reported experimental observations of crack blunting and delayed crack propagation as a result of lower *R*.^52^

**Figure 2:**
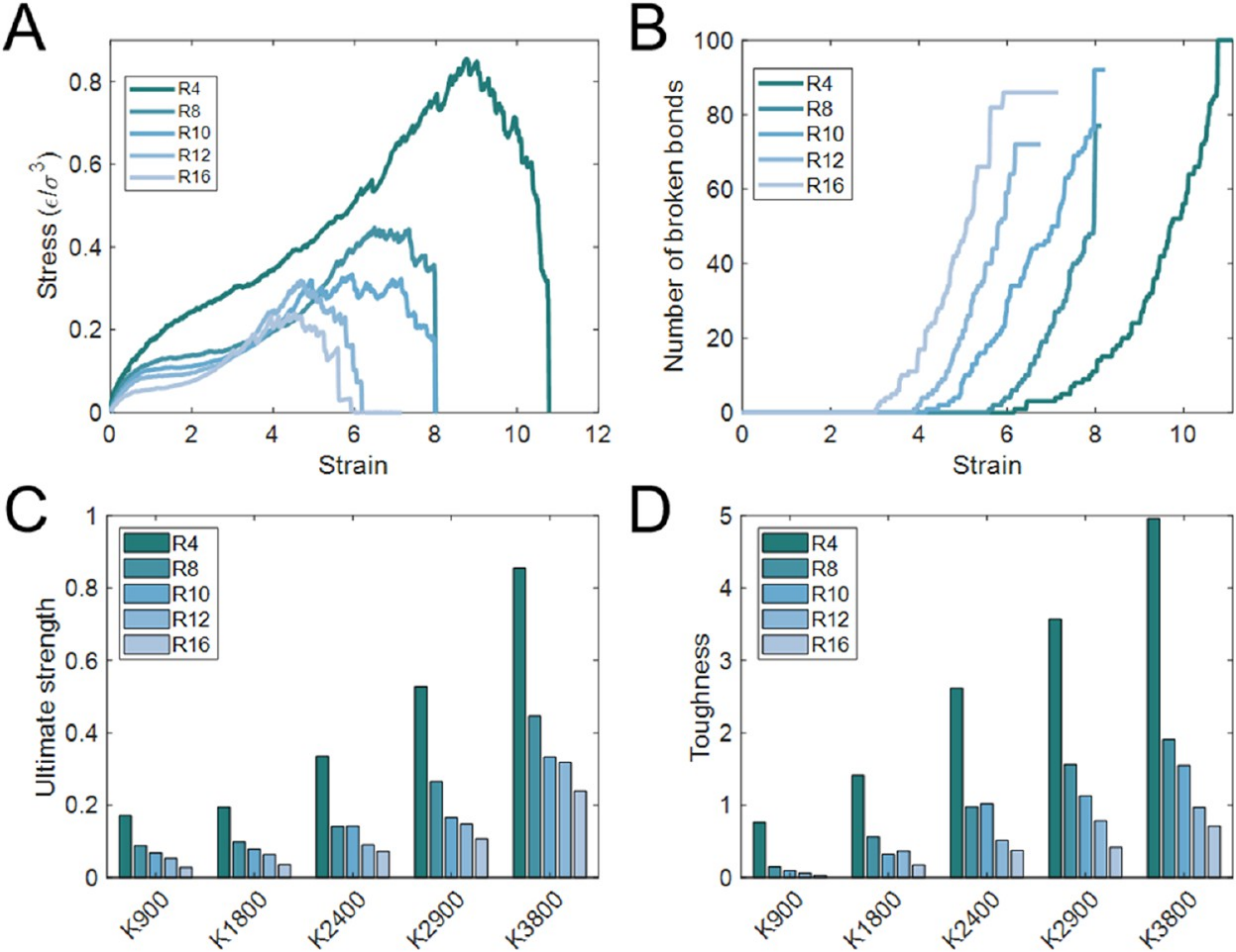
Mechanical response of SR networks with *N* = 40 and *K* = 3800 at different *R*. (A) Stress–strain curves for networks with *R* = 4–16, showing reduced peak stress and earlier failure at larger *R*. (B) Number of broken bonds as a function of strain, indicating delayed bond rupture in networks with smaller *R*. (C) Ultimate strength and (D) toughness as a function of *K* for different *R*. Both properties decrease with increasing *R*. The values of (C, D) are averages over three independent simulations, and the minor error bars indicate standard deviations.

**Figure 3:**
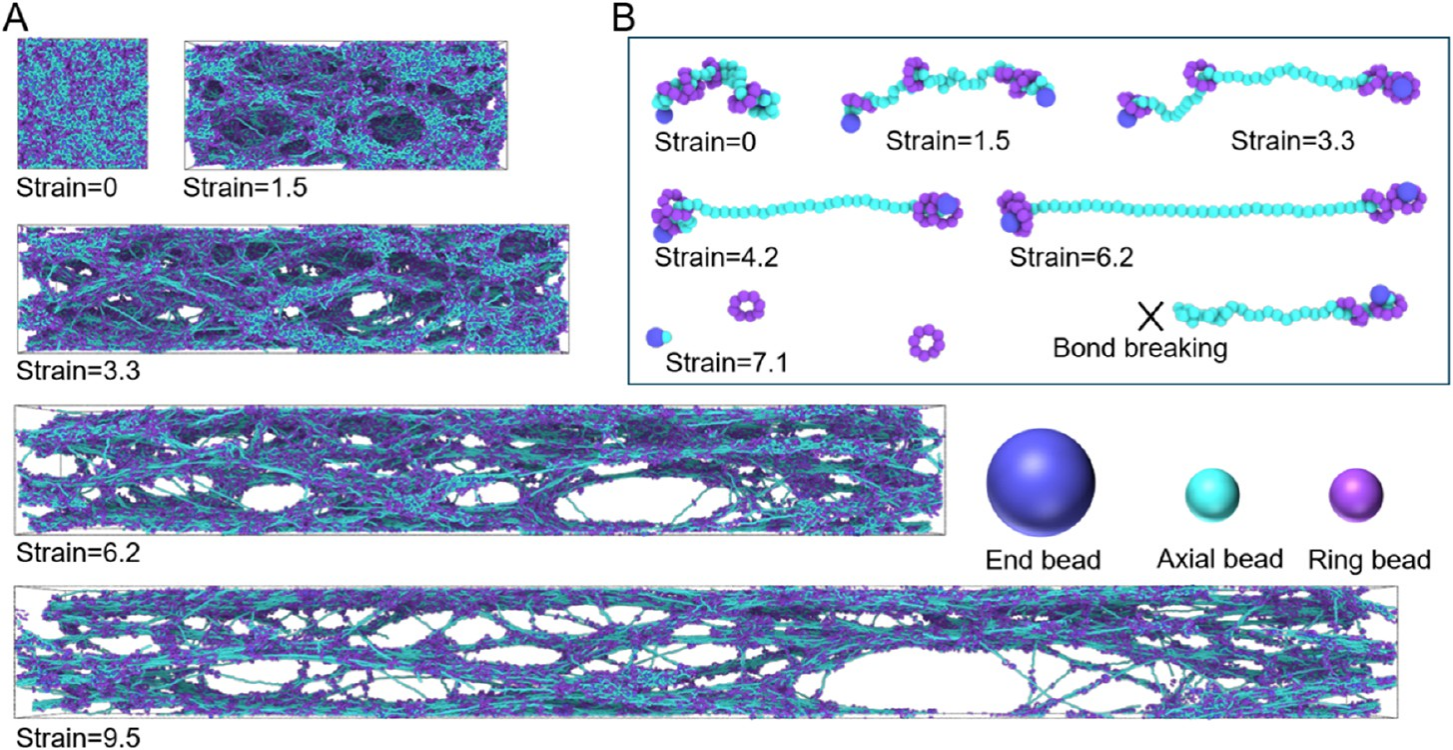
Structural evolution of N40R4K3800 networks under uniaxial stretching. (A) Snapshots of network evolution at increasing strain, showing void nucleation, growth, and coalescence, accompanied by progressive chain alignment. (B) Representative chain configurations at different strain, illustrating chain reorientation along the tensile axis, ring migration toward the axial chain ends, and eventual bond rupture. Beads are colored according to type: larger chain end beads (blue), axial beads (cyan), and ring beads (purple).

The ultimate strength (maximum stress) and toughness (area under the stress–strain curve) were further quantified for different *K*. As shown in Figure 2C,2D, both properties decrease monotonically with increasing *R*. At *K* = 3800, the ultimate strength reaches ∼0.86ε/σ^3^ for *R* = 4 but drops to ∼0.25ε/σ^3^ for *R* = 16, corresponding to nearly a 4-fold difference. The toughness shows a similar dependence, with values of ∼5.4ε/σ^3^ and ∼0.67ε/σ^3^ for *R* = 4 and *R* = 16, respectively. The trends are observed in the other cases with different *K* (Figure 2C,2D) and *N* (Figure S11). These simulation results are in good agreement with experimental observations, where higher ring density also leads to reduced strength and toughness.^49–52^

To study the effect of ring sliding, as a key mechanism, the ring distribution along the axial chains was analyzed. The configurations at both the undeformed and deformed states were selected for the system N40R4K3800 as an example. The center-of-mass of each ring was calculated and assigned to the nearest monomer along the same chain. Figure 4A,B show the distributions of these quantities of interests. As illustrated, at the initial undeformed state, the rings are randomly distributed between monomer indices 4 and 36 (Figure 4A). As the end groups are modeled as larger beads preventing ring detachment from the chain ends, consequently rings are seldom located in the chain ends (indices 1–3 and 37–40) at the initial state. Upon stretching, the number of rings in the middle region decreases while more rings accumulate at the chain ends (Figure 4B), as a result of the ring sliding. A representative structure is shown in Figure 4C, highlighting a single chain with rings concentrated near both ends. Similar behavior is also observed for cases with *R* = 8–16 (as shown in Figure S12). The distribution consistently evolves into a U-shaped profile with increasing strain. As the rings slide and accumulate near the ends, we further examined the spatial distribution of bond rupture along the axial chains (see Figure S13). The results show that bond-breaking events are concentrated at the chain ends, with relatively few breaks occurring in the middle of the axial chains. These findings indicate that ring sliding leads to stress concentration at the chain ends, promoting end-dominated bond rupture and failure. This mechanism is also consistent with recent simulation studies, showing that ring sliding redistributes tension toward the polymer chain ends, leading to localized stress concentration and subsequent bond rupture.^85^

**Figure 4:**
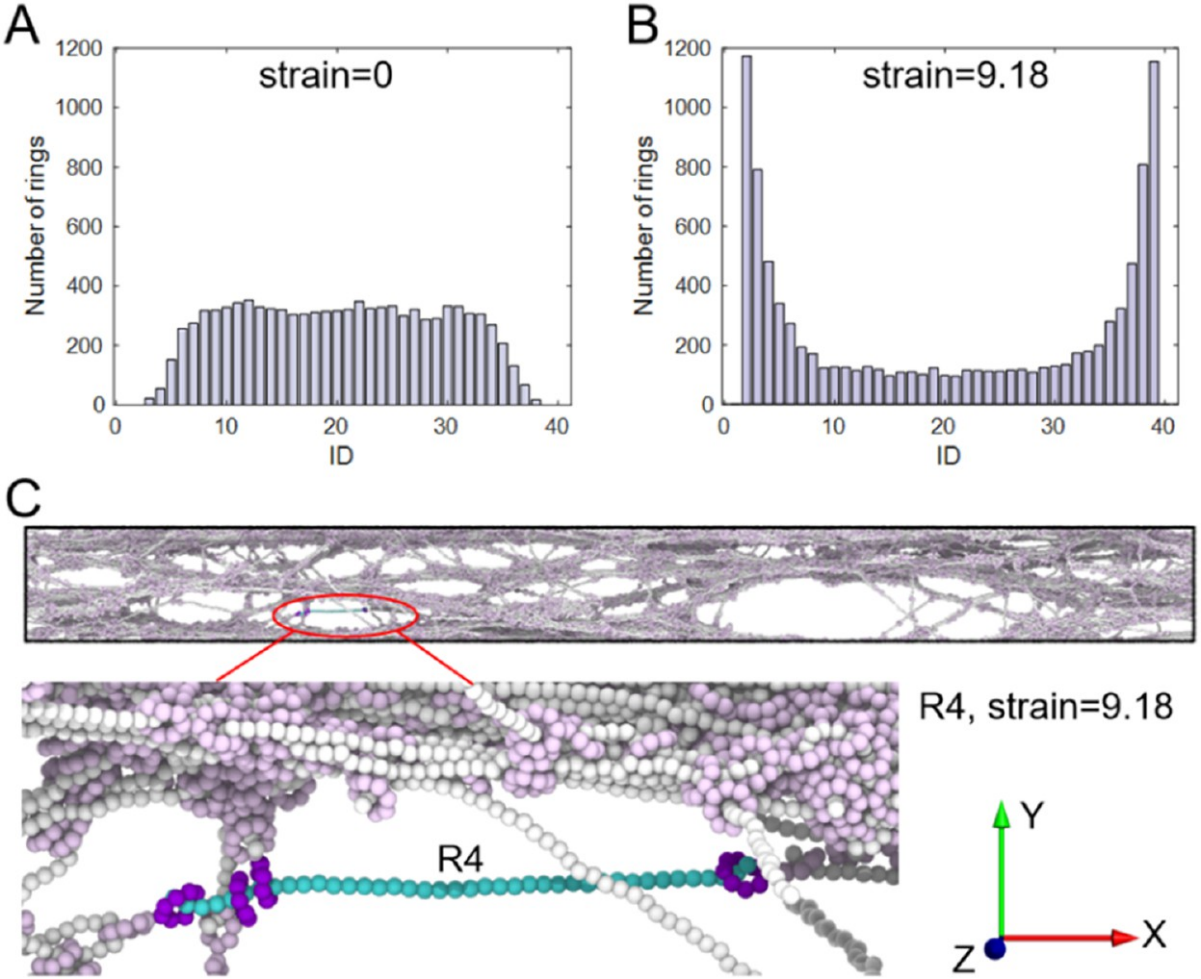
Distribution of rings along the polymer axial chains during stretching of N40R4K3800 case. (A, B) Distribution of ring centers of mass at the initial state (strain = 0) and the strain = 9.18. Rings are initially distributed throughout the axial chains but gradually migrate toward the chain ends, transforming from an inverted “U-shaped” to a “U-shaped” profile. (C) Representative structures illustrating a single chain at a large strain, where rings are concentrated near the chain ends, highlighting the sliding-induced ring accumulation at the ends.

Figure 4 demonstrates the occurrence of ring sliding, but it does not directly capture the *N*_slide_. Since the *N*_slide_ strongly influences the mechanical properties of SR networks,^46–48^ we further quantified it at different *R* (Figure 5). The *N*_slide_ was defined as follows: for the initial configuration (I), the center of mass of each ring was calculated and assigned to the nearest axial chain bead (*N*_I_). In the subsequent configuration (II), the center of mass was again located and reassigned to the closest axial chain bead (*N*_II_). The contour length of the axial chain between the two assigned beads was then taken as the $N_{\mathrm{slide}}=\sum_{N_{I}}^{N_{\mathrm{II}}}\left(L_{\mathrm{bond}}\right)$ . *L*_bond_ is the bond length between two adjacent beads within the range of *N*_I_ to *N*_II_. This procedure was repeated for all rings, and the average *N*_slide_ was calculated. Rings detached after axial chain bond rupture were excluded from the analysis. Since the axial chain bond lengths remain nearly unchanged during deformation (see Figure S14), the contour length of a chain is effectively identical whether calculated from configuration I or configuration II. As shown in Figure 5A for the system N40K3800 as an example, the average *N*_slide_ increases as *R* decreases. This trend can be rationalized by the fact that smaller *R* provides rings with an extended sliding domain along the axial chain (Figure 5B). The variation in sliding distance also leads to distinct deformation modes (Figure 5C). For R4, the network can sustain larger strains while still preserving its overall structural connectivity. However, for R16, the network nearly fails at a moderate strain of 5.7. Thus, reducing *R* increases the *N*_slide_, enabling the network to deform uniformly and remain connected at large strains, whereas larger *R* limits the ring sliding and causes premature failure of SR network.

**Figure 5:**
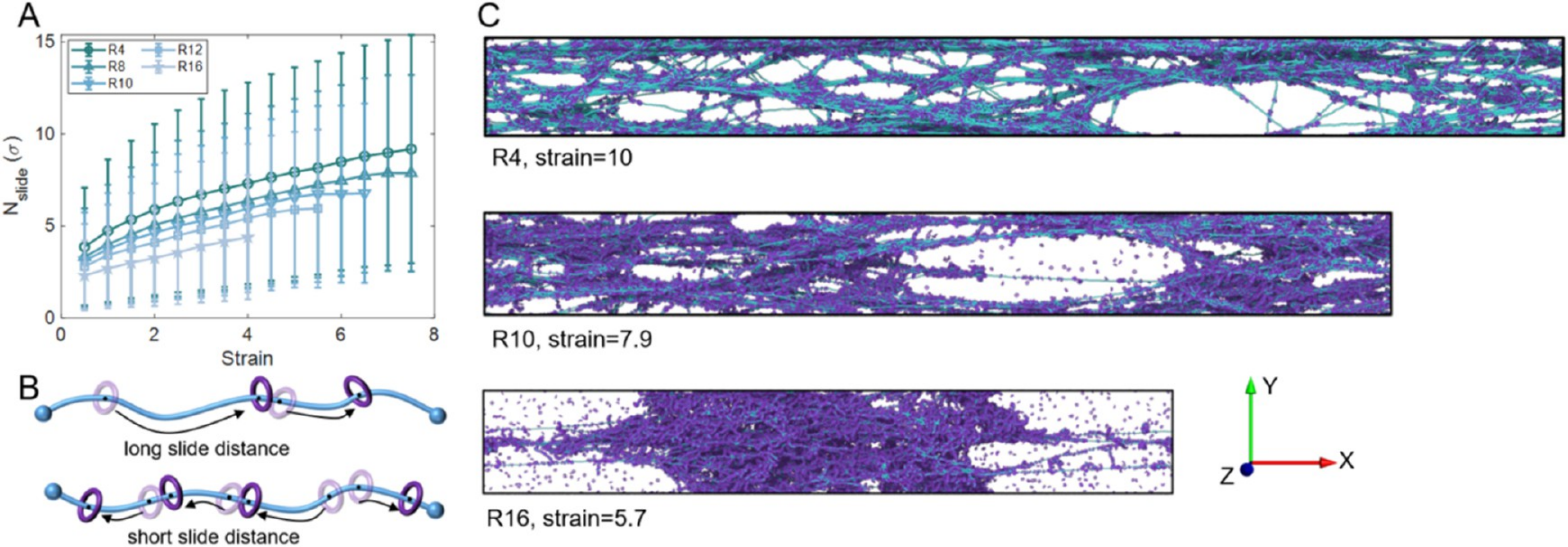
Effect of *R* on *N*_slide_ and network deformation at *N* = 40 and *K* = 3800. (A) The average *N*_slide_ with strain of different *R*. Error bars represent standard deviations. (B) Schematic illustration comparing long and short *N*_slide_ along the axial chain for different *R*. (C) Representative snapshots of network structures before failure for different *R*, showing that R4 sustains larger strains, whereas R16 fails prematurely.

From a mechanistic point of view, the deformation and failure modes described above represent the network-level response, whereas ring sliding also regulates the chain-level conformations. To elucidate the deformation mechanism, we evaluated the average order parameter, ⟨*P*⟩ of the axial chains along the stretching direction. This has been widely used to characterize chain orientation and conformational ordering.^85,96–100^

8 $$\left\langle P\right\rangle =\frac{3}{2}\left\langle \cos^{2}\theta \right\rangle -\frac{1}{2}$$

where θ is the angle between the vector connecting beads *n* – 1 and *n* + 1 and the strain direction (the *x*-axis). The bracket ⟨·⟩ denotes an average over all bonds along the axial chains. A value of ⟨*P*⟩ = 1 corresponds to complete alignment with the tensile direction, enabling the chains to sustain stress more effectively, whereas ⟨*P*⟩ = 0 indicates a random orientation.

As shown in Figure 6A, the initial values of ⟨*P*⟩ are close to zero, consistent with the isotropic chain conformations before deformation. ⟨*P*⟩ increases with increasing strain as the chains progressively reorient toward the tensile direction. The order parameter reaches a maximum before the network fractures, after which it decreases toward zero as the chains return to a random state. This trend is consistent with the structural evolution shown in Figure 3A (the postfracture structure is not shown for clarity). The polymer chains progressively stretch and reorient along the tensile direction, reflecting the alignment captured by the ⟨*P*⟩ analysis. Importantly, SR network with fewer rings attains higher maximum values of ⟨*P*⟩, and the corresponding strain is also larger. For instance, in the case of *R* = 4, ⟨*P*⟩ reaches a maximum of ∼0.6 at a strain of ∼11, whereas *R* = 16, the maximum ⟨*P*⟩ is only ∼0.35 and occurs much earlier, at a strain of ∼6. This indicates that reduced *R* allows chains to stretch and align more effectively along the loading direction, thereby delaying rupture and enhancing ductility and toughness of SR networks. These results are also consistent with the trends observed in Figure 2.

**Figure 6:**
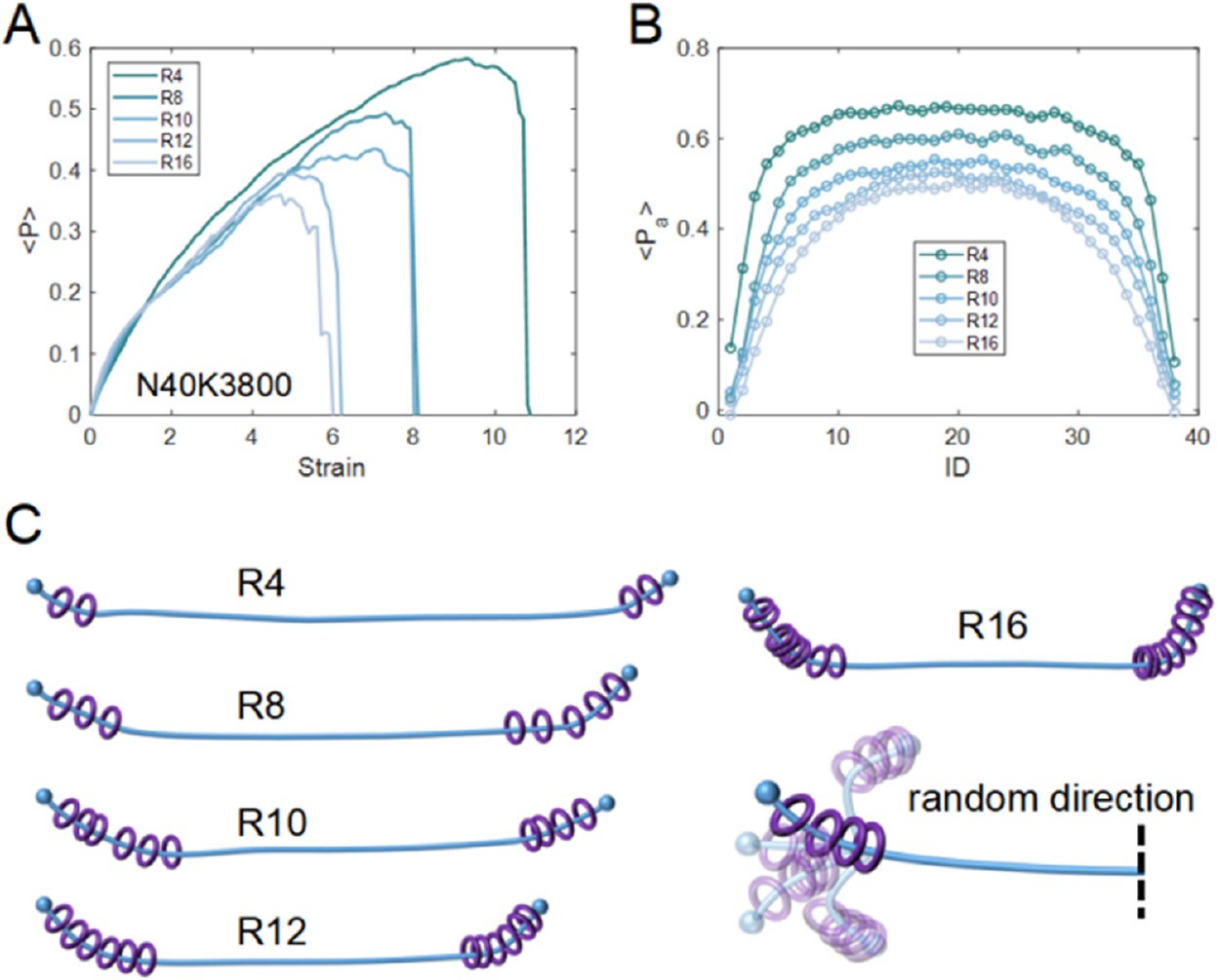
Chain orientation during deformation. (A) The average order parameter ⟨*P*⟩ for networks with different *R* = 4–16 at *N* = 40 and *K* = 3800. The results show higher maximum alignment at smaller *R*. (B) Atomic order parameter ⟨*P*_a_⟩ as a function of bead index, averaged over chains. The results indicate that orientation is highest at the axial chain center and decreases toward the ends, with stronger suppression at larger *R*. (C) Schematic illustration of rings’ positions, where rings progressively accumulate near the chain ends. Increasing R reduces the orientation of chain ends and then the overall orientation of the axial chains.

To further investigate the local response along the axial chains, the local order parameter ⟨*P*_*a*_⟩ were also evaluated for individual beads, defined as the average of ⟨*P*⟩ over different chains for the same bead index. The analysis was specifically performed for configurations corresponding to the maximum ⟨*P*⟩, so that the chain conformations could be more clearly resolved. As shown in Figure 6B, ⟨*P*_*a*_⟩ is highest near the middle of the chains and decreases toward the chain ends, where it approaches values close to zero. This behavior arises because the axial chains are modeled without angular constraints, allowing chain ends to adopt random orientations (Figure 6C). The results suggest that, during stretching, the rings tend to slide toward the chain ends, thereby preventing the end segments from effectively reorienting along the tensile axis and reducing ⟨*P*_*a*_⟩. Moreover, as *R* increases, the values of ⟨*P*_*a*_⟩ at corresponding positions along the chain become lower. This indicates that at larger *R*, more rings accumulate near the chain ends. As a result, the effective axial chain length that can align along the stretching direction becomes shorter, leading to reduced overall chain orientation. A schematic illustration of this mechanism is provided in Figure 6C, showing that ring accumulation at the chain ends reduces the level of overall chain-alignment, whereas fewer rings have a weaker influence on the overall chain orientation.

The structural evolution shown in Figures 3A and 5C indicates that varying *R* results in different failure modes as well as void formation and evolution. For example, as the strain increases, microscopic voids begin to form within the SR network, which subsequently grow and coalesce into larger cavities (Figure 3A). Understanding the formation and evolution of voids is essential for elucidating the fracture mechanisms of polymer networks.^96,101^ To gain deeper insight into this process, we focus on the evolution of voids during deformation. The simulation box was discretized into cubic cells of size 6 × 6 × 6σ^3^. The size of the cells ensured no voids in the initial state and effectively filtered out small fluctuations, allowing a clearer visualization of larger voids during deformation process. A cell was considered occupied if it contained at least one bead of the polymer network; otherwise, it was counted as a void. Neighboring void cells were grouped into a single connected void. And the total void volume was calculated as the number of void cells multiplied by the cell size. For computational convenience, we analyzed the void distribution using a single configuration at the selected strain and extracted the total void volume fraction, the average void size, and the maximum void size. Results from additional trajectories were provided in Figure S15 to demonstrate the robustness. This approach has been widely employed in the analysis of internal defects in polymer networks^96,101^ and biomaterials.^102,103^ Figure 7 illustrates the evolution of the total void volume fraction, the average void size, the maximum void size, and the void size distribution during uniaxial stretching for the representative case of N40K3800. As *R* increases from 4 to 16, the total void volume fraction increases (Figure 7A), indicating that the overall amount of internal defects formed during stretching is increased. From Figure 7B,C, it can be seen that at small strains, both the average void size and the maximum void size show little variation. At this stage, voids tend to nucleate randomly as small cavities within the network. With further deformation, however, small voids progressively coalesce into larger ones, which then expand outward as the primary damage sites, as given in Figure 7E. Consequently, at large strains the maximum void size increases almost linearly with strain. Moreover, in the intermediate strain range, both the average and maximum void sizes increase with *R*, suggesting that larger *R* facilitates void growth and accelerates failure, thereby reducing toughness of SR networks. At larger strains (strain >5), the networks progressively fail (also seen in Figure 2A,2B). Bond breaking leads to ring detachment and random diffusion of polymer beads, which causes strong fluctuations in the average void size.

**Figure 7:**
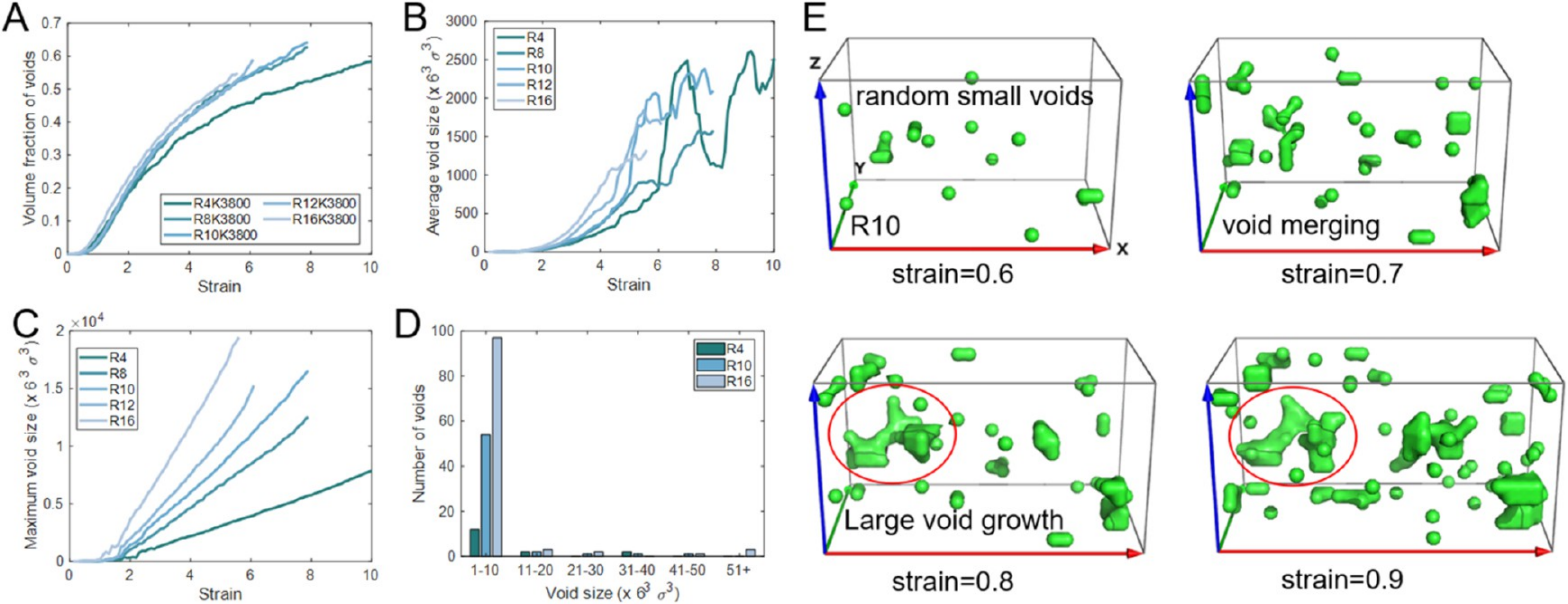
Quantification of void evolution for SR networks with *N* = 40, *K* = 3800, and *R* = 4–16. (A) Total void volume fraction as a function of strain, showing a reduction with decreasing *R*. (B) Average void size and (C) maximum void size. (D) Void size distribution at strain = 1 for networks with different *R*. (E) Representative snapshots of void evolution for the *R* = 10 system, illustrating the progression from randomly nucleated small voids at small strain to coalescence and growth into larger voids that expand with further deformation. For clarity, the polymer matrix is hidden and only the voids are displayed.

The void size distribution at strain = 1 was shown in Figure 7D. For *R* = 16, a large number of small voids (<20 cells) are generated, with nearly 100 voids smaller than 10 cells, also presented in Figure 8. Representative structural and void snapshots at strain = 5 are provided in Figure S16. In addition, a few larger voids (>40 cells) are also observed. By contrast, in the *R* = 4 system, fewer than 20 voids with sizes below 10 cells are detected, and only a limited number of medium-sized voids (31–40 cells) are observed, while no large voids (>40 cells) are formed. These results indicate that reducing the number of rings suppresses the formation of early stage defects, promotes more uniform deformation, and ultimately enhances toughness.

**Figure 8:**
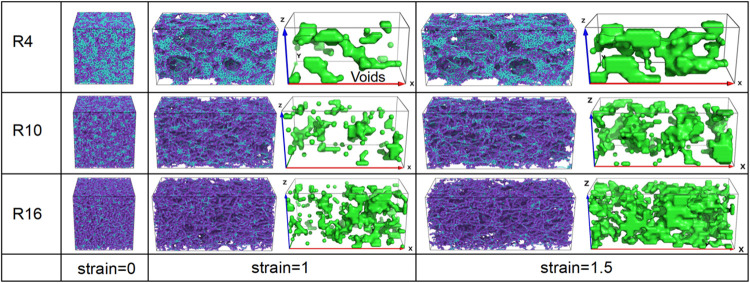
Representative snapshots of void morphology during deformation for networks with *N* = 40, *K* = 3800, and *R* = 4, 10, and 16. Snapshots are shown at strains of 0, 1, and 1.5. Green regions denote voids. Fewer and smaller voids are observed for *R* = 4, whereas for *R* = 16, it promotes the formation and coalescence of larger voids. Beads are colored according to type: chain end beads (blue), axial beads (cyan), and ring beads (purple).

### 3.2 Effect of Axial Chain Length

The mechanical properties of SR networks are dictated by *N*_slide_ of rings during deformation,^29,46,48^ which can be modulated through *R* or *N*. The influence of *R* has been discussed in the previous section, and we next examine the role of *N*. Figure 9A shows the stress–strain curves for SR networks with different *N* at *R* = 8 and *K* = 1800. With increasing *N*, the ultimate strength rises from 0.0984 (N40) to 1.5915 (N200), representing nearly a 16-fold increase. The toughness also increases from 0.5626 to 8.5465, corresponding to a 15-fold enhancement. For the short-chain system (N40), bond rupture occurs at a strain of 9 (Figure 9B), and the network has almost completely failed. Before this, the network undergoes progressive structural degradation and gradually loses connectivity, where the stress gradually decreased. This indicates that chain scission is not the primary failure mechanism for short chains; instead, chain pullout dominates, leading to progressive network failure before rupture. Chain pullout refers to a failure mechanism in which polymer chains were extracted or slide out of the network without covalent bond breaking. In contrast, for longer chains (N100 and N200), bond breaking initiates at strains above 7, accompanied by a significant stress increase. In these long-chain systems, chain pullout is difficult to occur, and chain scission thus becomes the dominant fracture mechanism.

**Figure 9:**
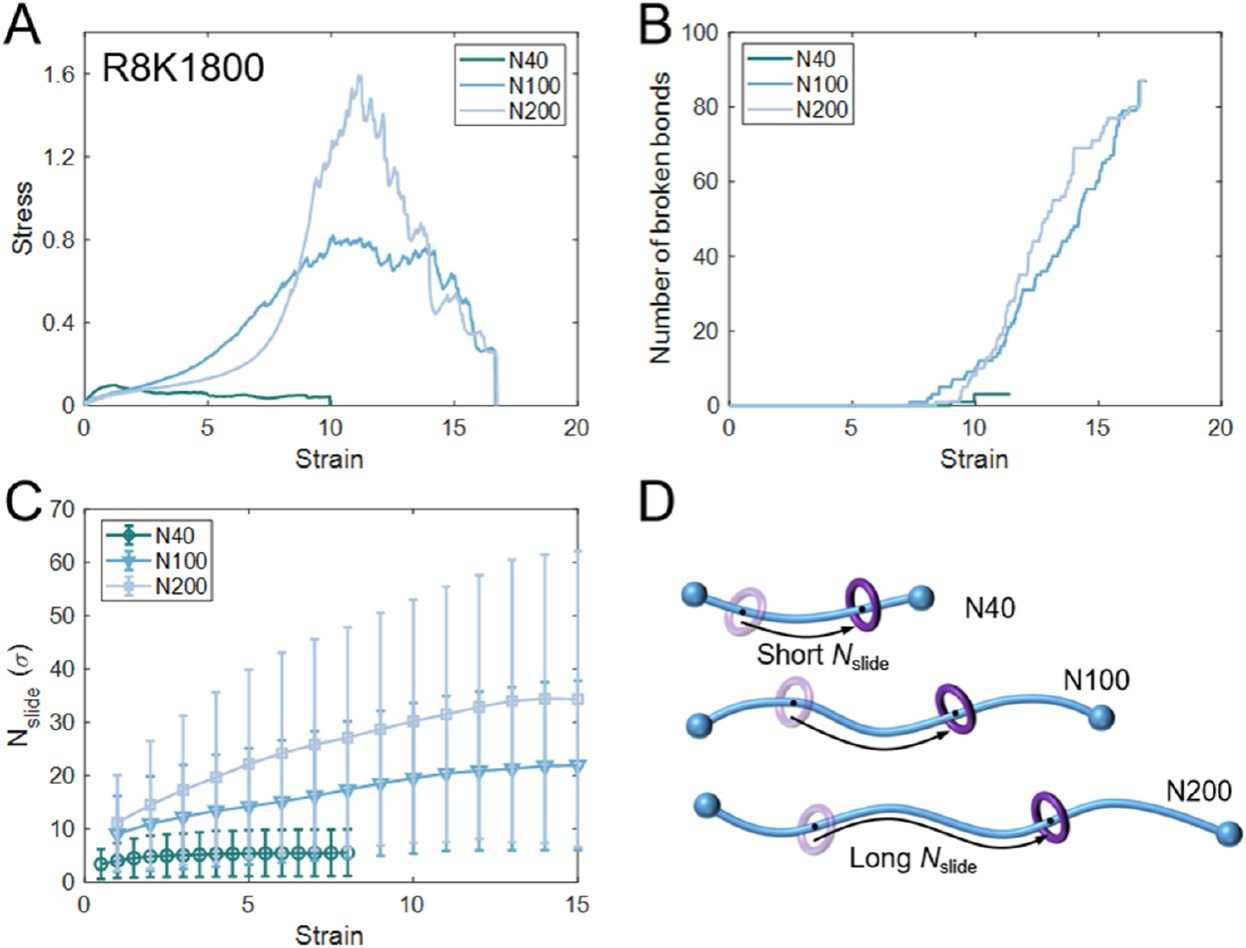
Effect of *N* on mechanical response, bond breaking, and sliding distance. (A) Stress–strain curves for networks with different axial chain lengths (N40, N100, N200) at *R* = 8 and *K* = 1800. (B) The number of broken bonds with strain. (C) Average *N*_slide_ with strain for different *N*, with error bars representing standard deviations. (D) Schematic illustration of *N*_slide_ for short- (N40), medium- (N100), and long-chain (N200) systems.

At strains below 7, networks with longer chains also show delayed stress increases, which is attributed to the allowable *N*_slide_ (Figure 9C,D). The increase of *N*_slide_ facilitates chain reorientation and relaxation of local tension. For example, the N200 case shows the most softened response as it has the largest *N*_slide_. Additionally, the order parameter of longer-chain systems increases more slowly, allowing chains to align at larger strains (as shown in Figure S17). Moreover, the maximum order parameter in longer-chain networks (N200) is higher, reflecting an enhanced capacity for chain alignment and thereby delaying fracture of SR networks. This mechanism can help explain recent experimental observations showing that long-chain αCD-based hydrogels (molecular weight of 10 kg/mol) display a slower stress increase at the small-strain stages than the short-chain counterparts (3.4 kg/mol).^58^

Notably, the N200 network reaches a higher ultimate strength than N100. This suggests that additional mechanisms are involved, most notably the emergence of entanglements in longer chains. Entanglements emerge as a form of topological constraint, as illustrated in the inset of Figure 10. Both experimental^104,105^ and computational^106,107^ studies have demonstrated that entanglements enhance the strength and toughness of polymer networks. To quantify this effect, the average number of entanglements per chain was analyzed using the Z1+ algorithm.^108^ As shown in Figure 10, the number of entanglements initially increases with strain and subsequently decreases. The network failure at large strain is accompanied by bond-breakage, chain-scission, and disentanglement, which damages the integrity of the SR network. For example, at strains above 7, the N100 and N200 systems gradually disentangle, and the decrease in entanglement correlates with the bond breaking, leading to a sawtooth-shaped stress response with alternating drops and rises. Notably, the N200 network maintains a higher number of entanglements than N100, which contributes to its greater strength. Thus, longer chains introduce stronger topological constraints, allowing the network to bear higher stresses, delay fracture, and ultimately enhance toughness.

**Figure 10:**
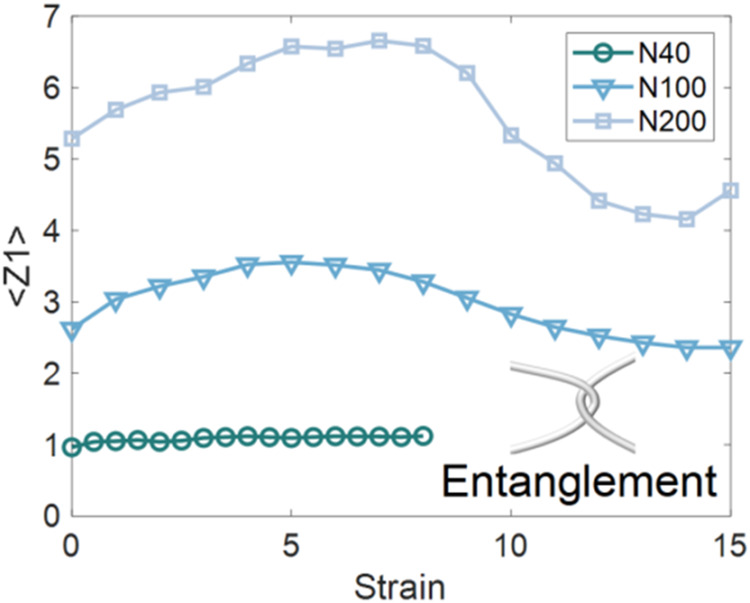
Evolution of the average number of entanglements per chain (⟨*Z*1⟩) as a function of strain for R8K900 networks with *N* = 40, 100, and 200. Longer chains exhibit a initial larger number of entanglements. Upon stretching, the number of entanglements initially increases, followed by a decrease at large strain due to disentanglement and progressive network failure. The inset provides a schematic illustration of an entanglement as a topological constraint.

As discussed above, increasing chain length also alters the failure mode. Figure 11A presents representative network configurations before failure at different strains. On one hand, for short chains (N40), failure is dominated by chain pullout, making the network unable to sustain large deformations. In this case, voids nucleate at relatively small strains and lead to failure. On the other hand, long-chain systems form craze-like structures during stretching, which dissipate a large amount of energy and thereby enhance network toughness. The presence of entanglements in long chains stabilizes these crazing structures. Importantly, we also observe that during early stage sliding, rings can encounter entanglements, which in some cases restrict further sliding (see Figure 11B). Consequently, ring sliding softens the network at small strains, whereas entanglements harden the network at large strains. This provides a mechanistic explanation for the stress–strain behavior shown in Figure 9A. In particular, longer chains have larger *N*_slide_, resulting in lower stresses at small strains, while their more entanglements enables them to sustain higher stresses at large strains.

**Figure 11:**
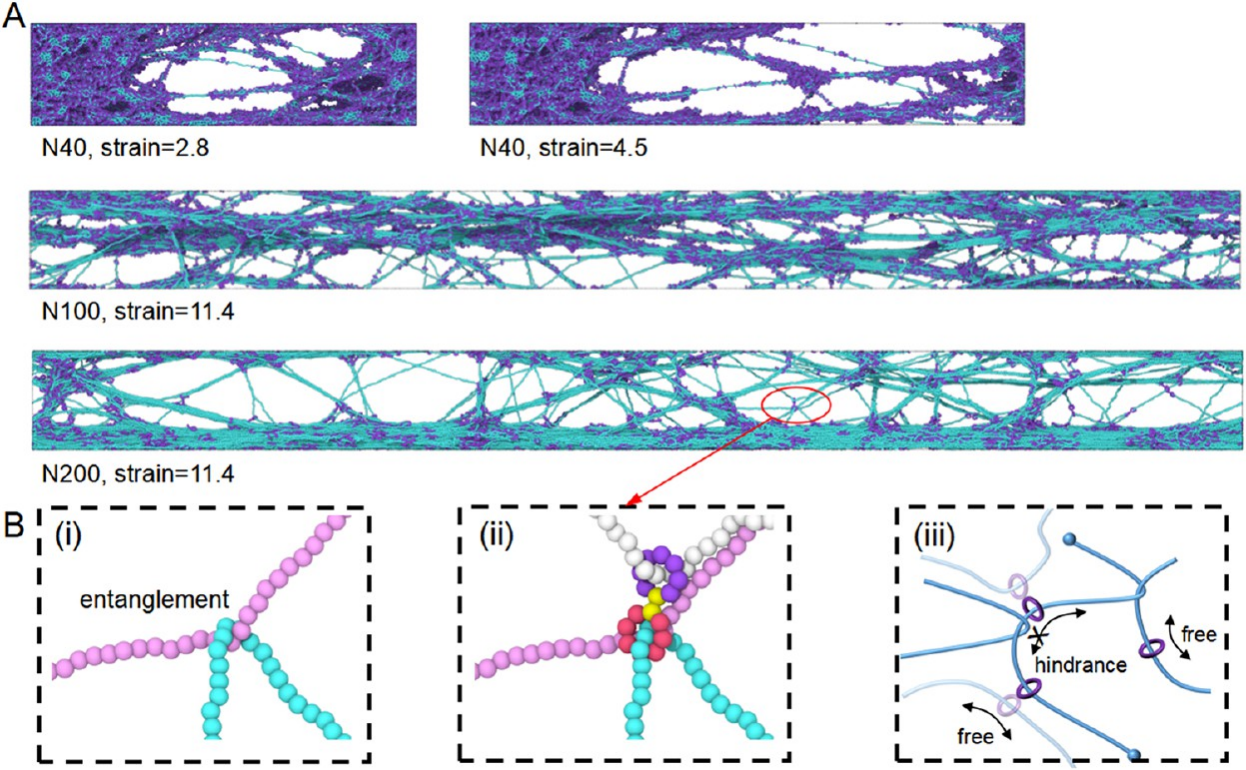
Structural evolution of R8K900 with different *N*. (A) Representative snapshots of network configurations before failure. Short-chain systems (N40) fail primarily through chain pullout and void formation (strain = 2.8, 4.5), whereas longer chains (N100 and N200) form craze-like structures at higher strains (strain = 11.4), which dissipate energy and enhance toughness. (B) The entanglement and the influence on ring sliding. (i) A representative entanglement between axial chains. (ii) Local snapshot of an entanglement region, as highlighted in N200. (iii) Schematic illustration showing that rings may slide freely along the axial chains. But sliding can also be hindered when encountering entanglements, thereby modulating the deformation response.

### 3.3 Effect of Cross-Link Number

Having understood the influence of *R* and *N*, we next examined the effect of *K*. Taking the case of *N* = 100 with *R* = 12, *K* was varied from 900 to 3800. Figure 12A,12B show the stress–strain curves and the corresponding number of broken bonds. At the early stage of deformation (strain <5), SR networks with more cross-links exhibit stiffer response, indicating that highly cross-linked networks can carry larger stresses at the same strain. As the strain increases to approximately 5–10, bond rupture initiates in the axial chains for all cases. Within this strain, the stress response exhibits accelerated growth, with pronounced fluctuations corresponding to successive bond breaking. After attaining the peak, the stress decreases to zero as the network undergoes complete failure. Although highly cross-linked networks sustain larger stresses, they also fail earlier. For example, the *K* = 3800 network reaches a maximum stress of ∼1.4 ε/σ^3^ but completely fails at a strain of ∼10. In contrast, the *K* = 900 network reaches a lower maximum stress of ∼0.3 ε/σ^3^ but survives until a strain of ∼17, indicating greater stretchability. The bond-breaking profiles (Figure 12B) further reveal that increasing the number of cross-links not only advances the onset of rupture, but also increases the total number of broken bonds. Similar trends were observed in systems with different *R* at *N* = 40, 100 and 200, as shown in Figures S18–S24. For *N* = 200 (Figures S22–S23), reducing *K* led to lower stresses but delayed failure, indicating enhanced stretchability. This behavior is consistent with that observed for *N* = 100. However, the *N* = 40 systems showed a different response: highly cross-linked networks not only carried larger stresses but also fractured at later strains, as seen in Figure S18. As discussed earlier, short-chain systems predominantly fail via chain pullout. Increasing *K* makes the network more continuous and promotes more homogeneous deformation, thereby enhancing stress transfer between different chains. Consequently, the networks can withstand higher stresses and strains. The structural evolution was further illustrated in Figure S19.

**Figure 12:**
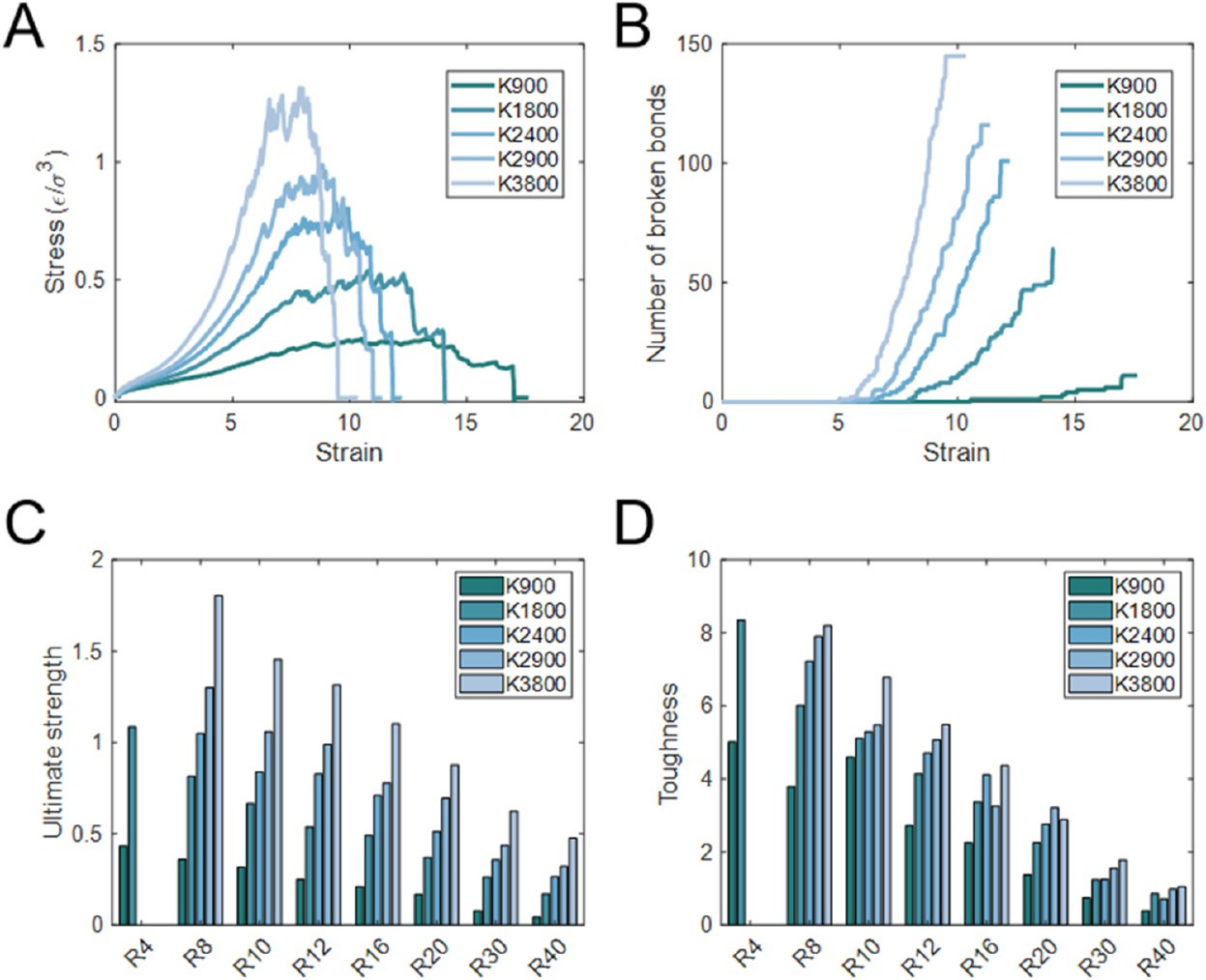
Mechanical response of SR networks with *N* = 100 and *R* = 12 at different *K*. (A) Stress–strain curves for networks with varying *K* from 900 to 3800, showing increased peak stress and earlier failure at larger *K*. (B) Number of broken bonds as a function of strain, indicating delayed bond rupture in networks with a smaller *K*. (C) Ultimate strength and (D) toughness as a function of *R* for different *K*. Both properties increase with increasing *K*. The values of (C, D) are averages over three independent simulations, and the minor error bars indicate standard deviations.

We also examined the effect of *K* on *N*_slide_. As shown in Figure 13A, all systems reach a similar maximum *N*_slide_ of 19σ upon deformation. This is expected, since the average *N*_slide_ is primarily determined by *R* and *N*. However, a key difference emerges: systems with larger *K* reach the maximum *N*_slide_ at smaller strains. The difference is attributed to the fact that the un-cross-linked rings slide more slowly than the cross-linked ones, as the latter bears the load. With increasing *K*, a greater fraction of rings participates in rapid sliding (Figure 13B). Thus, it enables larger *N*_slide_ at smaller strains. Consequently, due to the load-bearing effect, highly cross-linked networks can sustain larger stresses at early deformation stages (Figure 12A). The chain conformation also changes earlier, as reflected by the higher order parameter values at the same strain (see Figure S25). Moreover, a slight increase in the maximum order parameter is observed as *K* increases. This effect arises from the enhanced sliding of cross-linked rings, which facilitates the reorientation of a larger fraction of chains. Furthermore, bond rupture also occurs earlier in highly cross-linked systems, leading to premature network failure (Figure 12A,12B). This mechanism can help explain the experimental trend observed in β-CD-based hydrogels, where increasing *K* leads to higher strength, but reduces extensibility.^109^

**Figure 13:**
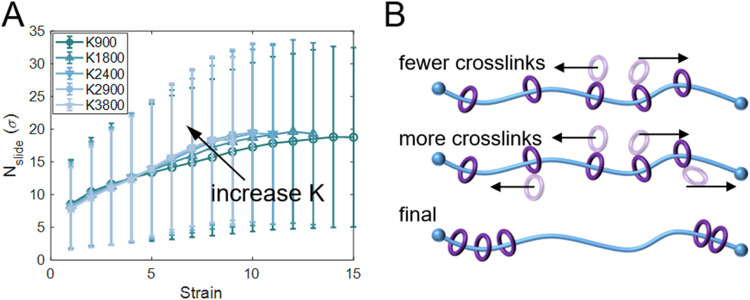
Sliding distance with different *K* at *N* = 100 and *R* = 12. (A) The average *N*_slide_ with strain for systems with different *K*. All systems eventually reach a similar maximum *N*_slide_, but larger *K* accelerate the approach to this limit. Error bars represent standard deviations. (B) Schematic illustration of ring sliding under different cross-links. In SR networks with fewer cross-links, only a limited number of rings slide faster. In contrast, networks with more cross-links contain a larger fraction of rings that slide more rapidly, enabling the system to reach its maximum *N*_slide_ earlier.

To directly quantify the load-bearing role of rings underlying this dragging effect, we further analyzed and compared the atomic stress of cross-linked and non-cross-linked rings using the equivalent von Mises stress. This analysis was performed for the N100R12K3800 system at a strain of 7.5, where the high cross-link density results in many cross-linked rings and stress localization is more evident at large deformation. The results for the N100R12K900 system were provided in Figure S26. The von Mises stress is defined as $\sigma_{\mathrm{Mises}}=\frac{1}{\sqrt{2}}\sqrt{{\left(\sigma_{1}-\sigma_{2}\right)}^{2}+{\left(\sigma_{2}-\sigma_{3}\right)}^{2}+{\left(\sigma_{1}-\sigma_{3}\right)}^{2}}$ , where σ_1_, σ_2_ and σ_3_ are the principal stresses obtained from diagonalization of the atomic stress tensor. Additionally, once the polymer axial chain is broken, the stress is released. Therefore, all rings cross-linked to this chain are treated as non-cross-linked in the analysis. As shown in Figure 14A,B, cross-linked rings exhibited a higher average von Mises stress than non-cross-linked rings. Moreover, stress concentration was also observed on cross-linked rings and at nearby chain-end segments (Figure 14C,14D), highlighted by the red-colored beads. The accumulation of rings near chain ends further promoted stress concentration at chain ends, making these regions more susceptible to bond breaking. These results provide direct quantitative support for the dragging effect, in which cross-linked rings experience larger mechanical loading and therefore slide faster than non-cross-linked rings.

**Figure 14:**
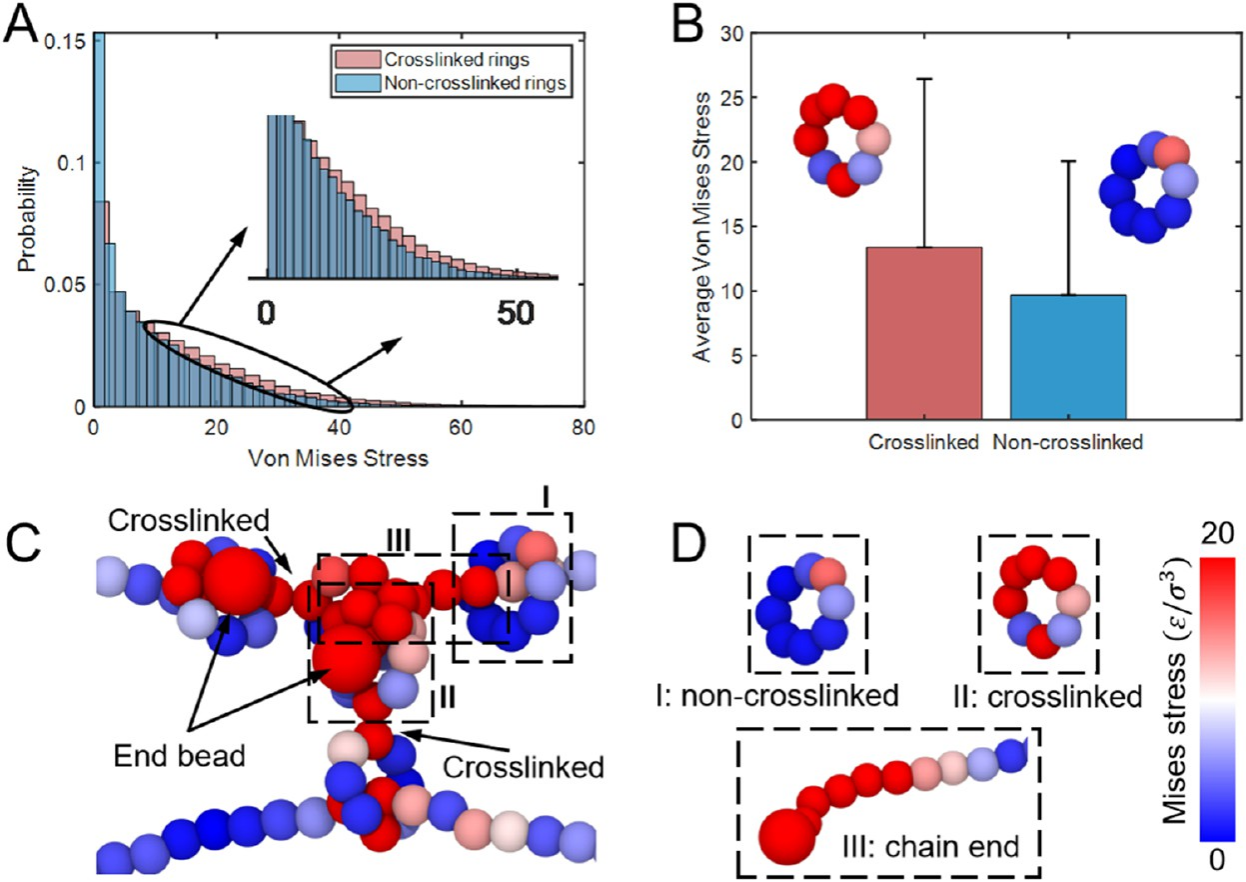
Local stress distribution and concentration in cross-linked and non-cross-linked rings for the N100R12K3800 system at strain = 7.5. (A) Probability distributions of von Mises stress of cross-linked and non-cross-linked rings. (B) Average von Mises stress for cross-linked and non-cross-linked rings, showing that cross-linked rings bear a higher load. Error bars represent standard deviations. The inset illustrates representative cross-linked and non-cross-linked rings colored by von Mises stress. (C) Representative snapshot illustrating stress concentration around cross-linked rings and chain-end beads. (D) The zoom-in views of the non-cross-linked ring (I), cross-linked ring (II), and chain-end segment (III). The color map in (C, D) represent the magnitude of the von Mises stress.

To further investigate the effect of *K* on toughness, the per-atom stress along the *x*-direction was evaluated. Two representative cases, *K* = 900 and *K* = 3800, are shown in Figure 15. In the initial state, the local stress distribution is relatively uniform (Figure 15A). With increasing strain, the stress distribution gradually shifts to the higher stress values, indicating an increase in tensile stress along the *x*-direction, particularly at stress >10. Meanwhile, the fraction of chains under compressive stress decreases, as evidenced by the reduction of negative stress contributions. The population around zero stress increases, reflecting the stress-relaxation effect facilitated by ring sliding. A similar trend is also observed in the N100R12K900 system, as shown in Figure S27. In the initial state, the atomic stress distributions of the *K* = 900 and *K* = 3800 systems are nearly identical (as shown in Figure S27). However, at strains of 3.75 (Figure S27) and 7.5 (Figure 15B), the highly cross-linked system (*K* = 3800) exhibits a rightward shift toward higher stresses, with a larger fraction of chains subjected to tensile loading. Representative structures in Figure 15C,D further illustrate that the *K* = 900 system develops only a few localized regions of high stress at strain = 7.5. The fewer stress localization delays bond breaking and thereby delays network failure. However, in the *K* = 3800 system, a large number of chains bear significant loads, as highlighted by the red segments in Figure 15D. Consequently, in the highly cross-linked network, chains under localized stress are more likely to rupture, leading to earlier failure of SR networks. Nevertheless, because the highly cross-linked system sustains much larger stresses before failure, the overall toughness still exhibits an increasing trend compared to weakly cross-linked systems.

**Figure 15:**
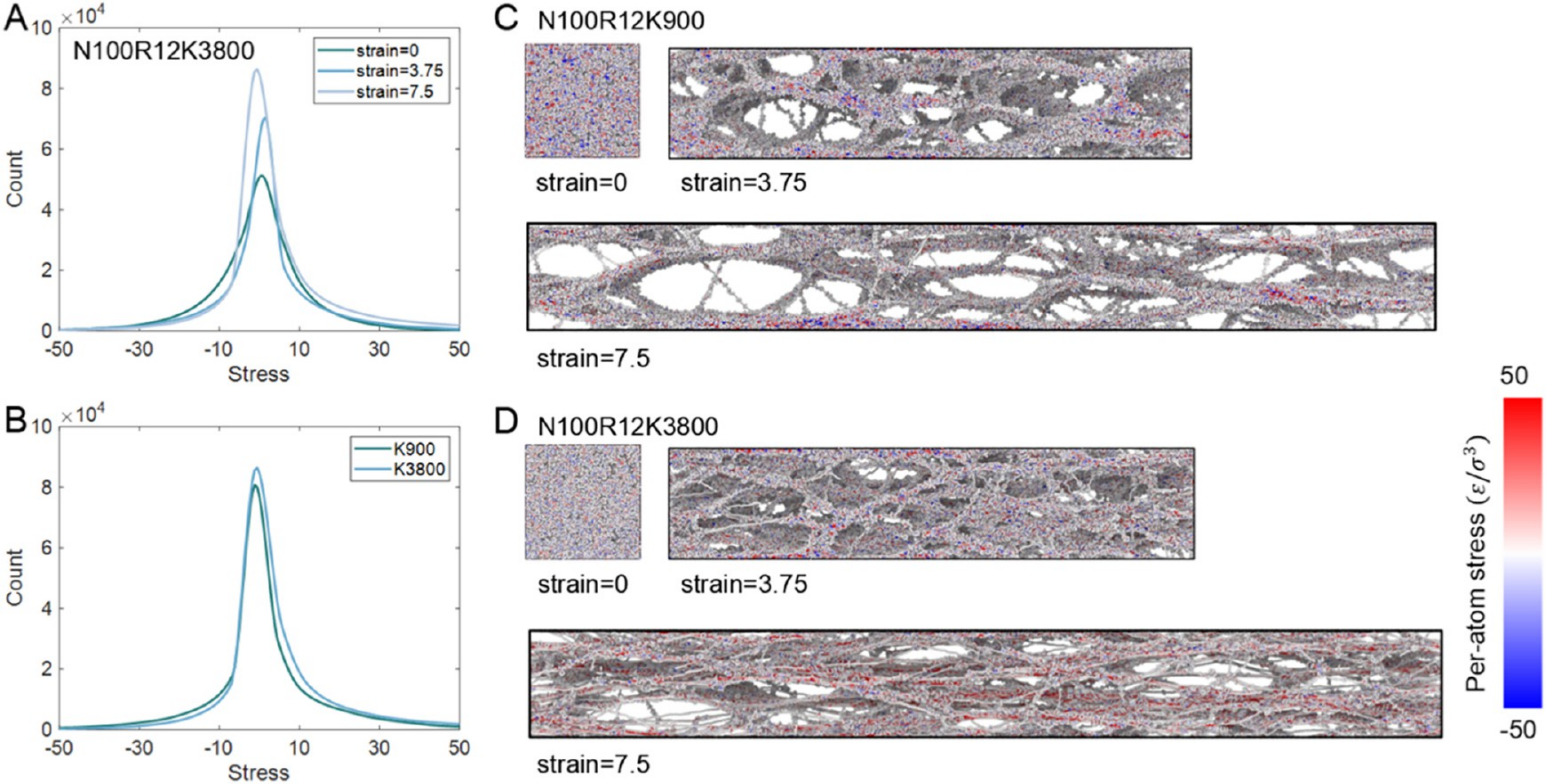
Local stress distribution along the stretching direction for SR networks with *N* = 100, *R* = 12, and different *K*. (A) Stress distribution for the *K* = 3800 system at strains of 0, 3.75, and 7.5. (B) Stress distribution for *K* = 900 and *K* = 3800 networks at strain = 7.5. (C, D) Representative snapshots of per-atom stress for *K* = 900 (C) and *K* = 3800 (D) at strains of 0, 3.75, and 7.5. The color scale indicates the per-atom stress in the *x*-direction. For small *K* (C), only a few localized regions of high stress emerge at large strain, whereas for large *K* (D), many chains carry significant loads, leading to stress concentration and earlier rupture.

From the structural evolution (as shown in Figure 15C,15D), it is also evident that *K* affects the evolution of voids, which directly reflects internal defects of the network. The total void volume fraction, average void size, and maximum void size, with representative snapshots of void evolution, are shown in Figures S28 and S29. With increasing *K*, the total void volume fraction, average void size, and maximum void size all decrease. Moreover, highly cross-linked systems generate fewer small voids. This void evolution indicates that deformation proceeds more homogeneously at a larger cross-link number, thereby enhancing the toughness of the network.

### 3.4 Overall Trends in Strength and Toughness

The combined effects of *R*, *N*, and *K* on the ultimate strength and toughness are summarized in Figure 16A. Across all systems, the minimum strength was 0.027 (N40R16K900), while the maximum reached 2.3 (N200R16K3800). Similarly, the toughness ranged from 0.033 (N40R16K900) to 11.19 (N200R4K900). As we discussed above, increasing *R* reduces both strength and toughness, whereas increasing the *N* and *K* enhances both properties. Consequently, the weakest networks can be easily found in systems with larger *R* and smaller *N* and *K* (N40R16K900). It should be noted that the *K* is influenced by *R*, since each cross-link is formed between a pair of rings. Although a larger *R* allows an increase in possible cross-links, it also decreases the overall strength and toughness, indicating a coupled effect. For instance, in the N200 system, the maximum toughness (11.19) was achieved at N200R4K900. The small *R* (R4) was limited to only 1000 possible cross-links (500 chains × 4 rings per chain ÷ 2). With fewer cross-links available, the network exhibits significantly reduced strength (0.907). By contrast, the maximum strength of 2.301 was observed for N200R16K3800. In this case, the larger *R* enabled the *K* to reach 3800, thereby enhancing the strength. However, the increased *R* also reduced the toughness of the network. Notably, the N200R12K2900 system exhibits both high toughness (10.327) and high strength (2.025). The relatively more rings (R12) ensures more cross-links (K2900), while *R* is not sufficiently large to markedly compromise the mechanical performance. Furthermore, short-chain systems (N40) exhibited relatively narrow distributions of strength and toughness. By contrast, longer-chain systems (N200) showed much broader variations, which reflect the increasing complexity of ring distribution, cross-link distribution, and entanglements. For example, the N200 systems exhibited strength values ranging from 0.312 (N200R40K900) to 2.3 (N200R16K3800), a ∼7-fold enhancement, while the toughness ranged from 2.672 (N200R40K900) to 11.19 (N200R4K900), a ∼4.2-fold increase.

**Figure 16:**
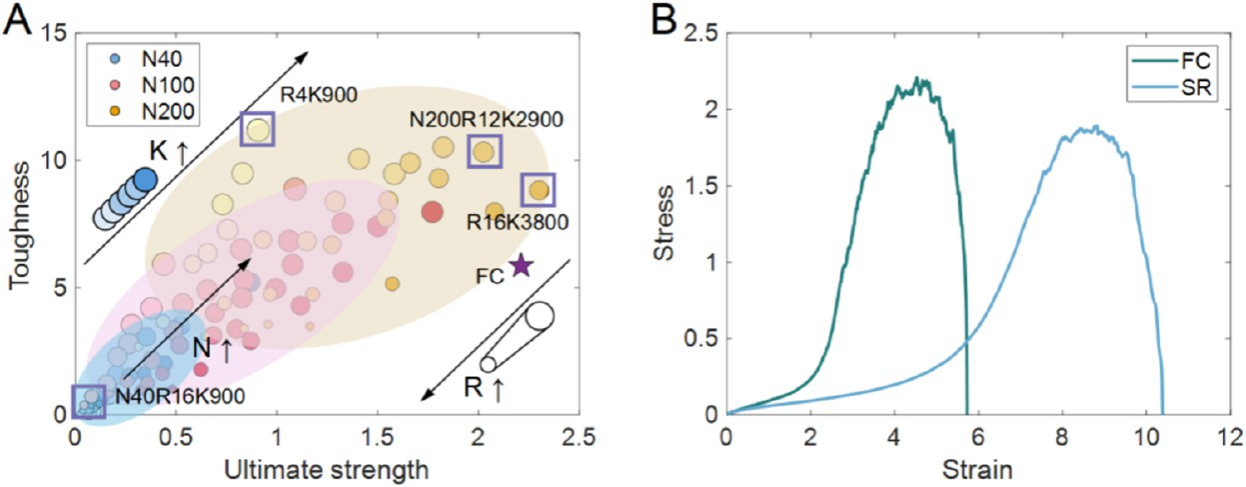
Effects of *R*, *N*, and *K* on the ultimate strength and toughness of SR networks. Each circle represents one simulated system, with colors distinguishing axial chain lengths (*N* = 40 in blue, *N* = 100 in red, *N* = 200 in yellow). Within each chain length, darker colors indicate larger *K*, while smaller circle sizes correspond to larger *R*. Arrows indicate the general trends: increasing *K* and *N* enhance both strength and toughness, while increasing *R* reduces both properties. Representative network cases are highlighted with boxes. (B) Stress–strain responses of FC and SR networks at N200R12K29000, in a large-scale system containing 5000 chains. The FC network exhibits larger ultimate strength but is subject to earlier failure. The SR network shows lower stress at small strains but sustains deformation to larger strains, demonstrating enhanced extensibility. The purple star in (A) denotes the FC result.

To assess the robustness of SR networks at larger scales, a representative system with both high strength and toughness (N200R12K2900) was expanded from 500 chains to 5000 chains. The results, compared against FC networks, are shown in Figure 16B. For the large system, the SR network maintained comparable strength (1.89) and toughness (7.1) to the smaller system. Although the FC network exhibited higher strength (2.2), its toughness (5.86) was lower than SR. Moreover, the fracture strain of the FC network (∼5.8) was substantially smaller than that of the SR network (∼10.2), highlighting the delayed fracture characteristic of SR networks. These observations are consistent with recent experimental and theoretical reports, that SR networks enhanced the toughness and delayed the fracture.^47,66,85,110^

## 4 Discussion

To elucidate the effect of *R*, we analyzed the spatial distribution of rings and quantified their sliding during tensile deformation. The *N*_slide_ was used to characterize the sliding distance. Ring sliding alters chain conformations and relaxes local tension, thereby enhancing the mechanical performance of SR networks. Our results show that decreasing *R* increases the sliding distance, which improves the ability of chains to reorient along the tensile direction. As a result, chains can more effectively bear stress and strain. The failure mode also shifts from void-dominated failure at small strains to craze formation at large strains, which dissipates significant energy and improves toughness. During deformation, sliding rings accumulate at chain ends, reducing the orientational order parameter near the chain ends and causing local stress concentration that ultimately triggers chain scission and progressive network failure. These findings are consistent with recent simulation studies: reduced *R* leads to ring accumulation at chain ends and consequently higher chain orientation, thereby improving the mechanical properties.^85^ Void analysis further confirms that decreasing *R* lowers the void volume fraction, average void size, and maximum void size, reflecting fewer internal defects and a delayed onset of failure. This molecular mechanism can also explain recent experimental observations. Reduced *R* leads to enhanced stress relaxation, delayed crack propagation,^52^ and higher ultimate strength and toughness.^49–53^ Kato et al.^51^ proposed a conceptual model in which ring accumulation near the chain ends under strain generates strong steric crowding and excluded-volume constraints. At larger R, this crowding significantly hinders further ring migration and suppresses chain extensibility, thereby reducing the mechanical performance. In agreement, our local order parameter analysis shows that larger *R* results in lower chain end orientation due to ring accumulation. At smaller *R*, the order parameter is higher, indicating that the rings can be further compressed. This additional compression allows greater chain stretching, leading to increased chain end orientation and enhanced extensibility of SR networks.

To investigate the effect of *N*, the analysis of *N*_slide_ and entanglements shows that ring sliding primarily softens the network at early strains, leading to lower stresses in long-chain systems. At larger strains, however, entanglements dominate the response. Longer chains can form stable craze structures, which dissipate large amounts of energy. Increasing chain length also increases entanglements and suppresses chain pullout, making chain scission the dominant failure mode. In addition to cross-links introduced by rings, entanglements provide additional topological constraints between chains, thereby enhancing both strength and toughness. This mechanism can help explain recent experimental observations of rotaxane-based hydrogels, showing that higher molecular weight polymer networks exhibit superior mechanical performance.^46,54–56,58^ This finding is also consistent with previous simulations on conventional FC polymer networks, where short chains primarily fail through chain pullout, whereas long chains fail through bond rupture-induced chain scission.^86,106,111,112^ Moreover, entanglements stabilize craze structures and enable a stable plastic deformation regime.^86,106,111,112^ In SR networks, however, ring sliding can be hindered by entanglements. Recent experimental results also indicate that the presence of entanglements hinders ring sliding and prevents complete network relaxation, thereby retaining a remaining elasticity.^113^ Here we propose two possible scenarios. In one case, rings are trapped at strong entanglements and cannot slide further. As deformation proceeds, axial chain bond rupture and disentanglement occur. This is unfavorable for ring sliding and limits network extensibility. In the other case, weak entanglements can slip,^89^ allowing rings to slide further together with the moving entanglement. This process, however, introduces additional intermolecular friction, which may also affect mechanical performance.^114^ Although both scenarios are possible, the overall response of SR networks is still dominated by ring sliding, since these systems are not highly entangled.

To investigate the effect of *K*, we first analyzed the *N*_slide_. The results show that the increasing *K* does not affect the maximum *N*_slide_, but rather the time required to reach it. In highly cross-linked systems, cross-linked rings slide more rapidly, whereas un-cross-linked rings slide more slowly. As a result, highly cross-linked systems transfer larger stresses at early strains, causing chains to enter the stretching regime earlier and leading to premature rupture. In addition, higher cross-link number improves network connectivity, suppresses void formation, and promotes more homogeneous deformation. These trends are consistent with recent simulation results of nanoparticle polymer composites, which also reported a reduction in void formation with increasing *K*.^115^ However, experimental studies have reported that in a β-CD-based polymer network, toughness initially increases with cross-link density and then decreases.^77^ Even though other results found the fracture energy of SR networks is less sensitive to cross-link density compared to FC networks, it still follows a similar trend of initial slight enhancement followed by reduction.^46^ We suggest that our current simulations are conducted at relatively low cross-link densities, such that increasing the cross-link number enhances toughness. However, in highly cross-linked systems, we hypothesize that a large fraction of ring sliding induces rapid configurational rearrangements. This, in turn, promotes pronounced premature fracture at small strains and reduces the toughness.

Recent simulations further found that larger *K* drives the distribution of sliding rings toward more extreme configurations, producing both shorter and longer chain segments during deformation.^66^ To test this, we calculated the distribution of segment lengths in N100R12 networks (Figure 17). A single chain with *R* rings is divided into *R*+1 segments (Figure 17A). We computed the contour length of each segment and analyzed its distribution. As shown in Figure 17B for *K* = 900, the initial distribution follows a single-exponential form, consistent with recent simulations^66,67,69^ and indicating that rings are randomly distributed along the axial chains.^66^ Similar results are obtained for other *K* systems as well, as shown in Figure S30. In our N100R12 system (*R*/*N* = 0.12), the most probable segment length is 3–4σ, in agreement with previous reports.^66,67^ Systems with similar ring density, such as N400R60 ^66^ (*R*/*N* = 0.15) and N200R28 ^67^ (*R*/*N* = 0.14), also show a maximum probability at 3–4σ. With increasing strain, ring sliding drives the segment length distribution to split into shorter and longer segments, yielding a biexponential profile. This behavior is consistent with previous simulations, which also reported a biexponential distribution of segment lengths.^66,67,69^ However, Figure 17C shows that increasing *K* in our simulations does not produce obvious changes in the segment length distribution, which contrasts with recent findings.^66^ They reported larger *K* led to more long segments and short ones.^66^ We attribute this discrepancy to the relatively small *K* used in our simulations. The average *K* per chain is only 0.9–3.8, compared to 7.5–25 in previous studies.^66^ Even when compared within the range of 7.5–10, the differences in segment length distributions remain small.^66^ Therefore, we conclude that our *K* is too low to capture this effect. Thus, increasing *K* in our systems enhances both strength and toughness, consistent with experimental observations that toughening occurs at low cross-link densities.^77^ However, increasing *K* is often accompanied by an increase *R*.^109,116^ In our systems, achieving larger *K* would require introducing more rings. This can be achieved in two ways: (1) Increasing *R* at the current chain length, but it inevitably reduces *N*_slide_; (2) employing longer chains and larger *R* with the same *R*/*N*, it maintains a comparable *N*_slide_. Therefore, the present study primarily reflects the low-cross-link-density regime, within which both strength and toughness increase with cross-link number.

**Figure 17:**
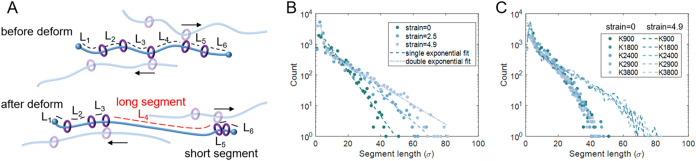
Segment length distribution in N100R12 networks. (A) Schematic illustration of how ring sliding during deformation redistributes segment lengths, leading to the formation of both short and long segments. (B) Segment length distributions at different strains with N100R12K900, showing a transition from a single-exponential profile to a double-exponential profile at larger strains. (C) Segment length distributions for systems with different *K* values. The distributions remain similar, indicating that cross-linking does not significantly alter the segment length distribution within the studied range.

## 5 Conclusion

In this study, we employed CGMD simulations to investigate the effects of ring number, axial chain length, and cross-link number on the ultimate strength and toughness of SR networks. A molecular mechanism of failure is proposed. Initially, rings are randomly distributed along the chains. Upon stretching, ring sliding induces chain rearrangement and progressive reorientation toward the tensile direction. Subsequently, the accumulation of rings near the chain ends leads to stress concentration, which triggers bond rupture at the axial chain ends. As rupture propagates, the network gradually fails. Importantly, the sliding distance of rings serves as a critical factor governing the mechanical properties of SR networks. Ring number per chain and axial chain length primarily govern the sliding distance of rings. Decreasing ring number and increasing chain length increase the sliding distance, thereby enhancing the ability of the network to reorient under strain. This effect enables the network to sustain larger deformations, delay final failure, and ultimately improve its mechanical performance. Although the number of cross-links does not affect the maximum sliding distance, it significantly influences the sliding dynamics. Cross-linked rings move more rapidly under strain, dragging un-cross-linked rings along. As a result, larger cross-link number systems accelerate ring accumulation at chain ends. This increases chain orientation and enables higher stress at small strains, but also leads to earlier fracture. Moreover, a higher cross-link number increases network connectivity. This allows stress to be transmitted more efficiently, resulting in more uniform deformation. However, the improved load transfer also causes stronger stress localization, leading to earlier fracture.

Specifically, increasing chain length not only extends the sliding distance, which alleviates stress concentration and slows stress buildup, but also, importantly, introduces interchain entanglements. These additional topological constraints, beyond the cross-linked rings, serve as a major toughening mechanism. We find that ring sliding primarily softens the network at early strains, while entanglements provide hardening at larger strains, together improving both strength and toughness.

Ring number, chain length, and cross-link number all changed the failure modes of SR networks. For smaller ring number, longer chains, or larger cross-link number systems, they promote the formation of stable craze structures, which effectively dissipate energy. Void analysis further reveals that both smaller ring number and larger cross-link number reduce the total void volume fraction, leading to more uniform deformation and improved mechanical performance. The molecular mechanisms underlying these effects differ slightly among the three parameters. For ring number, fewer rings increase the sliding distance, facilitating conformational changes and higher chain orientation, which enable more uniform deformation. For chain length, shorter chains tend to fail via pullout, creating weak contacts and void-mediated fracture. However, longer chains are constrained by additional entanglements, suppressing pullout and failing instead by chain scission. Therefore, it bears larger loads and deforms more uniformly. For cross-link number, the main role is to reinforce network connectivity and stress transfer, resulting in more homogeneous deformation under load. Overall, our findings highlight that the interplay among ring sliding, entanglements, and network connectivity governs the mechanical performance of SR networks. These results offer molecular-level insights into how structural parameters, such as ring number, chain length and cross-link number, affect mechanical behavior of SR networks. A clear understanding of these molecular mechanisms is therefore essential for the design of SR networks with tailored strength and toughness.

It should be noted that our simulations focus on moderate cross-link densities. The absolutely optimal conditions might differ in real systems with frictional or high cross-link density effects, but the mechanisms identified here should still hold. Our study can be extended to systematically explore the relationship between cross-link number and fracture toughness,^46^ exploring the high-density limit will be an important extension of this work. In such regimes, the interplay between ring sliding and the topological constraints of highly entangled systems is expected to become more significant.^113^ Moreover, the influence of strain rate on ring sliding dynamics has been observed experimentally,^47,48^ but molecular simulations can provide deeper mechanistic insights into how these dynamics govern the mechanical response. Additionally, incorporating specific ring–chain interactions, such as steric hindrance,^117,118^ electrostatic attraction,^119–121^ and hydrogen bonding,^122,123^ will be essential to capture more realistic sliding and failure behaviors. Addressing these aspects will be crucial for fully elucidating the fracture mechanisms of SR networks.

## Supplementary Material



## Notes

Details of the CGMD simulation: simulation setup details; convergence; network construction; mechanical response; bond rupture; ring sliding; chain alignment; bond length; structure evolution; local stress distribution; void evolution; and chain segment length distribution (PDF)
