# Supplementary material for: Molecular Mechanisms of Strength and Toughness in Slide-Ring Polymer Networks: Insights from Coarse-Grained Molecular Dynamics Simulations
Source: Macromolecules. 2026 Apr 3;59(7):3938–56. doi: 10.1021/acs.macromol.5c03515 (PMC13085803; doi:10.1021/acs.macromol.5c03515)
Supplement: Supplementary file 1 [file ma5c03515_si_001.pdf]

Supporting Information to

Molecular Mechanisms of Strength and Toughness in Slide-Ring Polymer Networks: Insights from Coarse-Grained Molecular Dynamics Simulations

Zihan Tang[1], Weikang Xian[1], and Ying Li[1*]

[1] Department of Mechanical Engineering, University of Wisconsin-Madison, Madison, Wisconsin 53706, United States

*Corresponding author: Email: yli2562@wisc.edu. Phone: 608-265-0577.

In the modeling stage, bond rupture was not considered and polymer chains were connected by finite extensible nonlinear elastic (FENE) bonds[1].

$$E_{\mathrm{FENE}}(r_{ij}) = -\frac{1}{2}kR_0^2\ln[1-(r_{ij}/R_0)^2] \tag{1}$$

where the parameters were $k = 30.0\varepsilon/\sigma^2$ and $R_0 = 1.5\sigma$ with a purely repulsive LJ potential, whose cutoff is $2^{1/6}\sigma$. The systems were simulated in the NVT ensemble at a reduced temperature of 1.0. Chain generation was initiated by randomly placing a bead within the simulation box, after which successive beads were added through a random-walk procedure to construct linear chains (**Figure 1A(i–ii)**). The initial density of system is $0.85/\sigma^3$. The systems were equilibrated for 5 x $10^6\tau$, followed by an additional equilibration of the same duration using the *fix bond/swap* algorithm in LAMMPS to accelerate relaxation[2]. The chain conformations after equilibration were assessed by calculating $\langle R_e^2\rangle/(nb^2)$, where $\langle R_e^2\rangle$ denotes mean-squared end-to-end distance of a chain segment containing n consecutive bonds[3,4]. And b=0.96σ is the average equilibrium bond length of the FENE potential. **As shown in Figure S1**, our results reproduce the expected increase of with segment length n and converge toward the characteristic ratio of approximately 1.8. Moreover, the three directional components (x, y, z) are nearly identical, indicating isotropic chain conformations. These observations confirm that the polymer chains were well equilibrated.

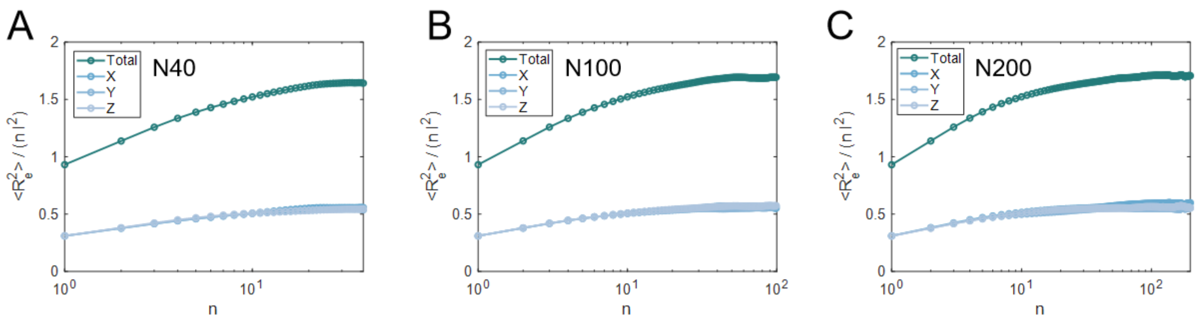

Figure S1. Characteristic ratio $\langle R_e^2\rangle/(nb^2)$ as a function of segment length n for chains with axial chain lengths of (A) N=40, (B) N=100, and (C) N=200. The total values increase with n and converge toward the characteristic ratio of ~1.8. The three directional components (x, y, z) overlap closely, indicating isotropic chain conformations.

Following equilibration, two types of systems were constructed. For the fixed crosslink (FC) networks, R beads along each chain were randomly selected as reactive beads, and intermolecular crosslinks were introduced to generate the network structure (**Figure 1A(iii)**). For the slide-ring (SR) networks, R beads per chain were chosen as anchoring sites for the attachment of eight-bead ring molecules, **as shown in Figure S2A**. The initial bond length of each ring was set to 0.6σ, and it was gradually increased by 0.1σ every 4.0 x $10^5\tau$ until reaching 1.0σ (**Figure S2A**). One bead on each ring was assigned as a reactive bead, and intermolecular crosslinking was subsequently performed over 3 x $10^6\tau$, yielding the SR networks (**Figure 1A(iv)**). After network formation, each system was equilibrated for 5 x 10^5 steps under NVT conditions followed by another 5 x 10^5 steps under NPT conditions with zero pressure. To prepare for subsequent fracture simulations, the bonded interactions were then switched to the quartic potential, and the additional 5 x 10^5 steps of NVT and NPT equilibration were conducted to obtain the final initial configurations.

For the SR networks, ring rigidity was maintained using harmonic bonds with a stiffness constant of $500\varepsilon/\sigma^2$. Angular constraints were also imposed to preserve the ring geometry: the eight internal angles were set to 135°, while the external angles formed during ring-ring crosslinking were set to 112.5° (**Figure S2B**). And all non-bonded interaction parameters were provided in Table S1[5]. The end beads are larger than the axial beads, leading to slightly weaker attractive interactions between the ends and the axial beads[5]. It can also prevent end-bead aggregation. Meanwhile, the LJ potential shift (Δr) between the rings and the end beads effectively suppresses ring detachment.

$$E_{\mathrm{LJ}}(r_{ij}) = 4\varepsilon_{ij}[(\frac{1}{r_{ij}-\Delta r})^{12} - (\frac{1}{r_{ij}-\Delta r})^{6} - U(r_{cutoff})] \quad (2)$$

Table S1. The non-bonded interaction parameters[5].

Pair		ε_{ij} (ε)	Δr (σ)	r_{cutoff} (σ)
Axial	Axial	1.0	0	2.5
Axial	End	5.0	0.5	2.24
Axial	Ring	1.0	0	$2^{1/6}$

End	End	1.0	1.0	$2^{1/6}$
End	Ring	1.0	0.56	$2^{1/6}$
Ring	Ring	1.0	0	$2^{1/6}$

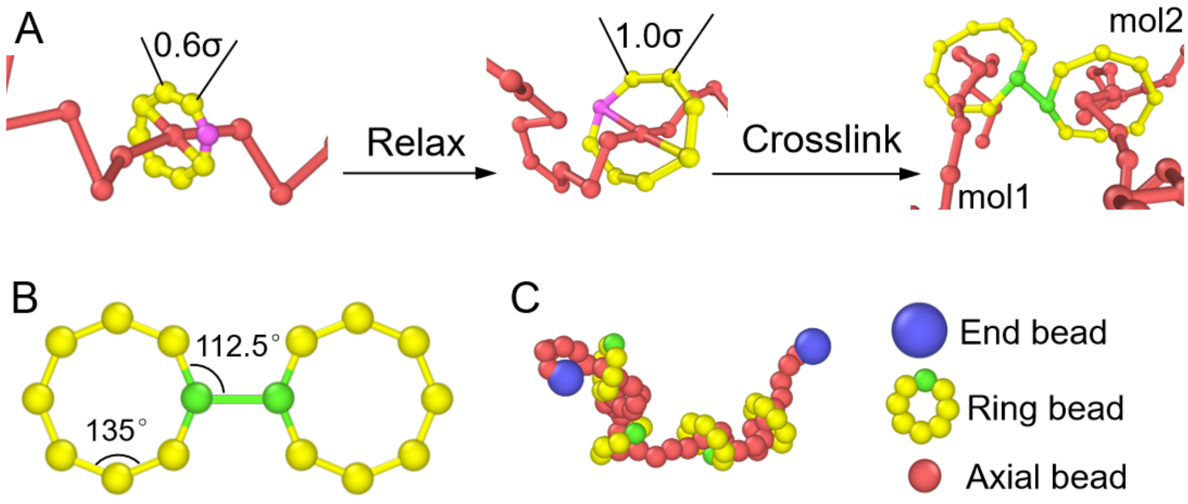

Figure S2. (A) Procedure for constructing SR networks. Rings initially attached with a bond length of 0.6σ were gradually relaxed to 1.0σ, after which intermolecular crosslinking was introduced between reactive beads on rings of different molecules. And the bonds between the ring and axial chains were then removed. (B) Angular constraints used to maintain ring geometry, with internal bond angles fixed at 135° and external angles formed during crosslinking fixed at 112.5°. (C) Schematic representation of the coarse-grained slide-ring chain, showing end beads (blue), axial beads (red), and ring beads (yellow and green).

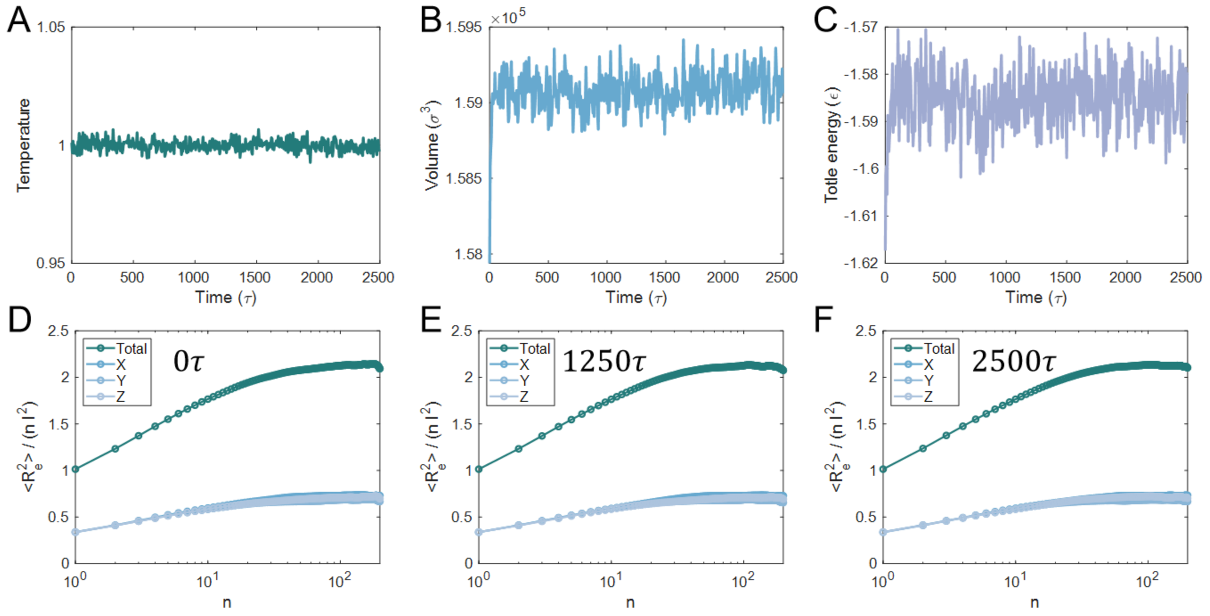

Figure S3. Equilibration of thermodynamic properties and axial chain conformations for the N200R8 system. (A-C) Time evolution of temperature, volume, and total energy during the last 2500τ of NPT equilibration, showing stable fluctuations around constant mean values. (D-F) Normalized mean-square end-to-end distance at three representative times: 0, 1250, and 2500τ. The nearly identical profiles converge to a plateau at large n and show no pronounced differences among the three spatial components (X, Y, Z), indicating that the backbone conformations were well equilibrated and isotropic.

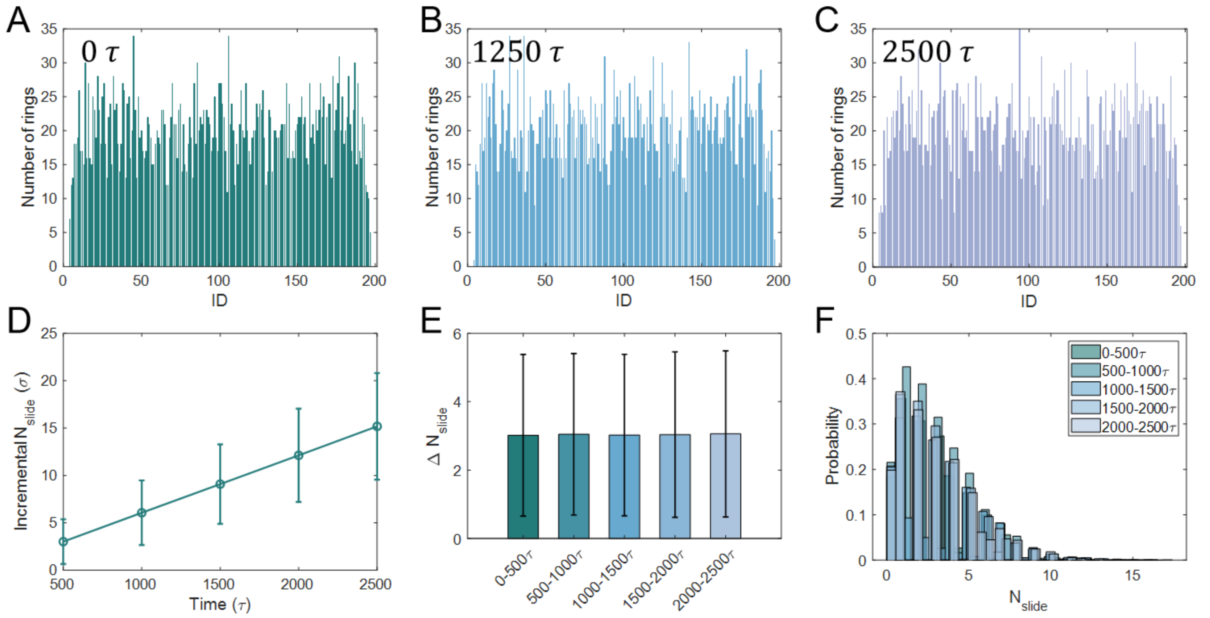

Figure S4. Time invariance of ring distribution and sliding statistics for the N200R8 system. (A-C) Distributions of the number of rings along the polymer axial chains at three representative times (0, 1250, and 250τ), showing random and statistically similar distributions. (D) Time evolution of the incrementally accumulated sliding distance. (E) Average sliding increment over successive 500τ intervals. (F) Probability distributions for the same time intervals. The similarity of the sliding increments and distributions indicates that ring sliding has reached a steady state.

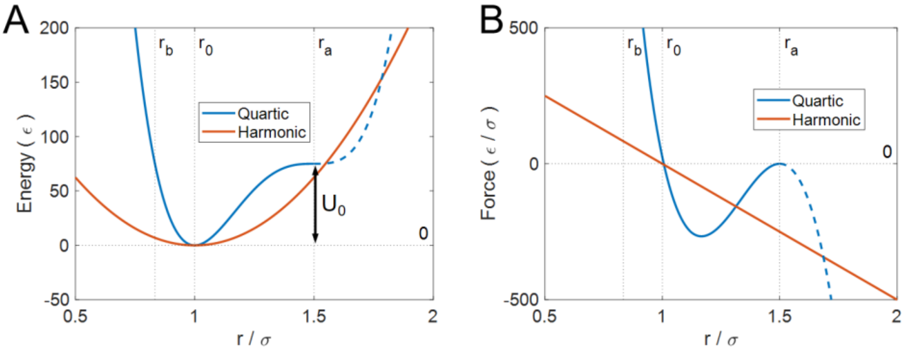

Figure S5. Comparison of quartic and harmonic bond potentials. (A) Bond energy as a function of bond distance r. ra is the cutoff distance beyond which the bond is removed, and U0 denotes the energy barrier. The parameter rb is selected such that the minimum of the quartic potential is located at r0=1.0σ, identical to the equilibrium bond length of the harmonic potential. (B) Force as a function of bond distance r.

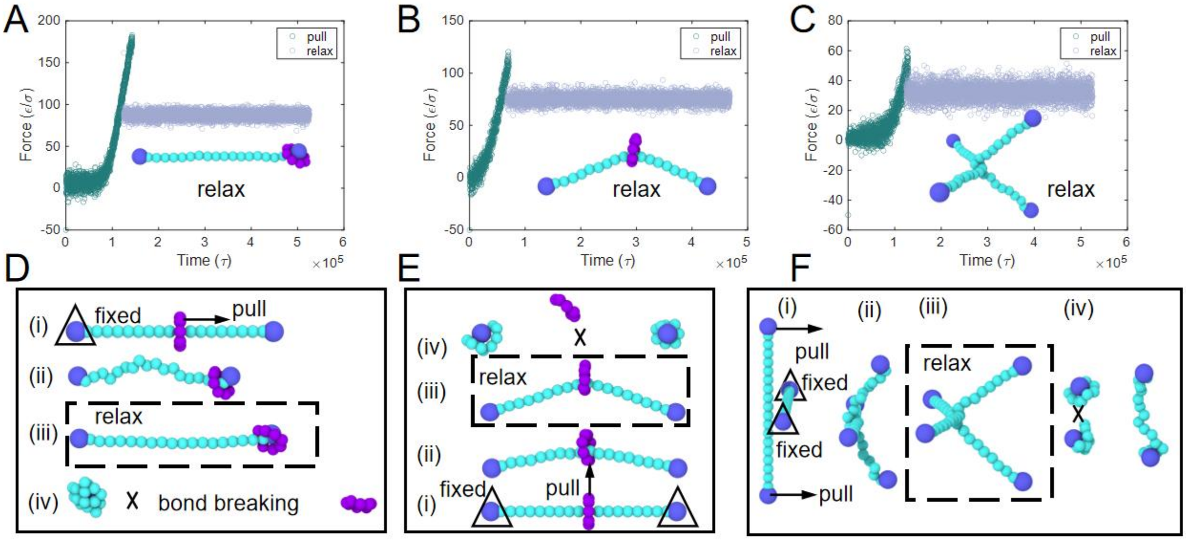

Figure S6. Tests for ring and entanglement passing under large deformation. (A-C) Force-time responses during pulling and subsequent relaxation for three representative loading geometries. Insets show representative relaxed configurations. (D) Schematic illustration of the ring un-stoppering test: (i)-(iv) ring pulled along the axial chain direction, where (iv) axial chain bond breaking (marked by X) occurs without the ring passing. (E) Schematic illustration of the ring crossing axial chain test: (i)-(iv) ring pulled perpendicular to the axial chain direction, where (iv) axial bond breaking (X) without ring passing. (F) Test for entanglement passing in a two-chain system without rings: (i)-(iv) both ends of one chain were pulled, where axial chain bond breaking (iv) occurred without entanglement crossing. The additional equilibration simulations were further performed, extracting the configurations before bond breaking in (iii) of (D)-(F). In all cases, ring passing and entanglement passing are not observed before bond breaking.

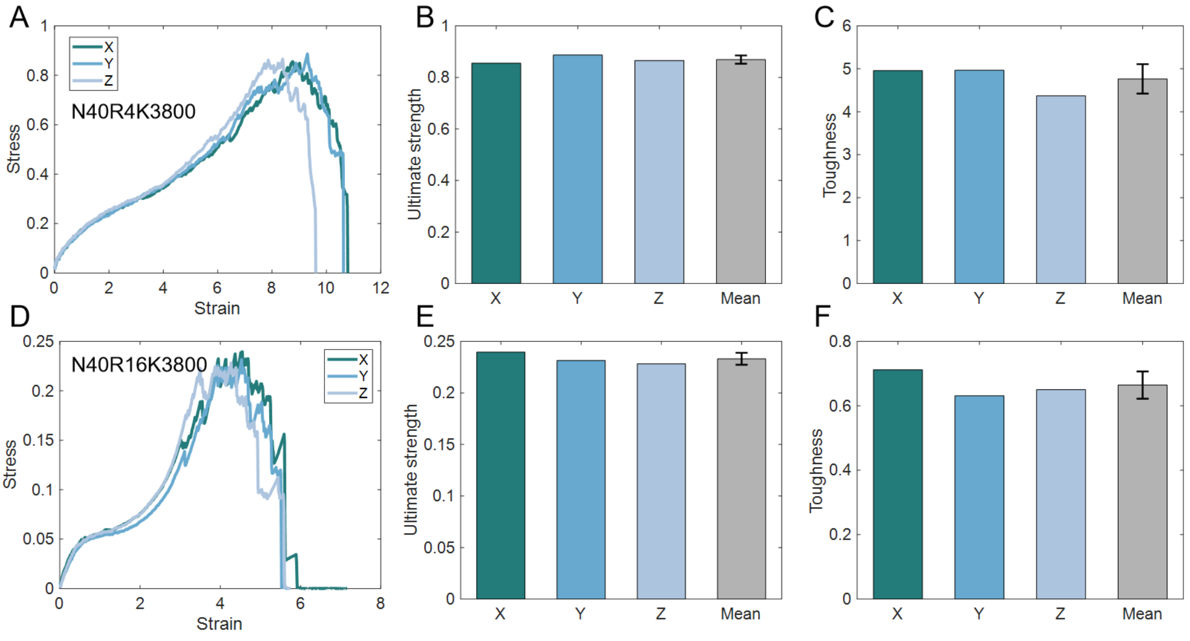

Figure S7. Verification of isotropy under different loading directions. (A-C) Stress-strain curves (X/Y/Z) and the corresponding ultimate strength and toughness for the N40R4K3800 system. (D-F) Same analysis for the N40R16K3800 system. The nearly overlapping stress-strain responses and the small variations in strength/toughness demonstrate an effectively isotropic mechanical response; error bars for the mean values represent standard deviations.

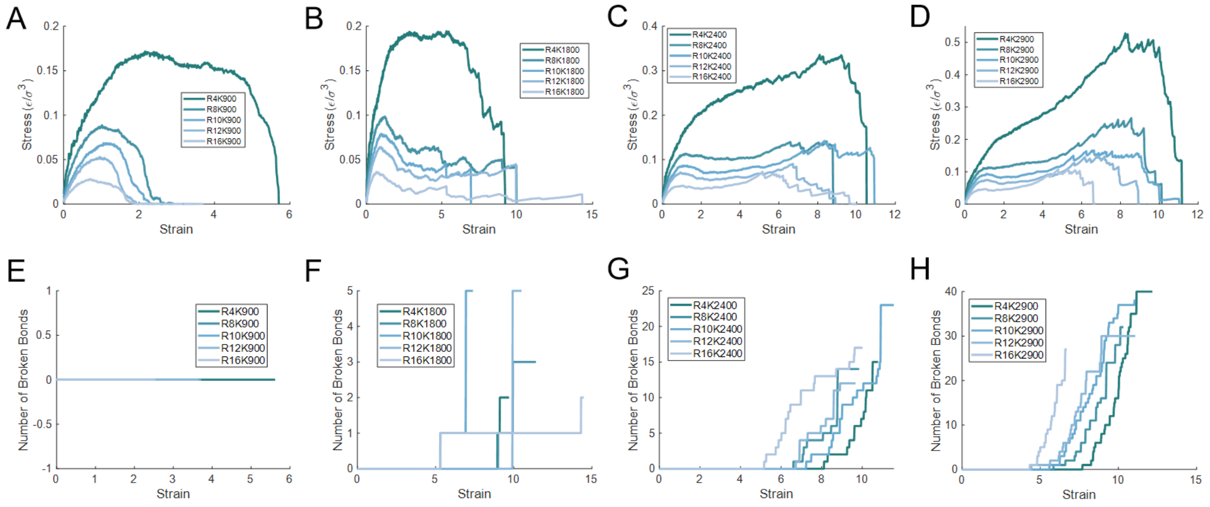

Figure S8. Stress-strain response and bond rupture behavior of networks with *N*=40 at different *K* and R. (A-D) Stress-strain curves for *K*=900, *K*=1800, *K*=2400, and *K*=2900, respectively, with *R* ranging from 4 to 16. (E-H) Corresponding bond rupture profiles for the same systems, showing that bond breaking initiates earlier with increasing *R*, whereas larger *K* increases the total number of broken bonds at failure.

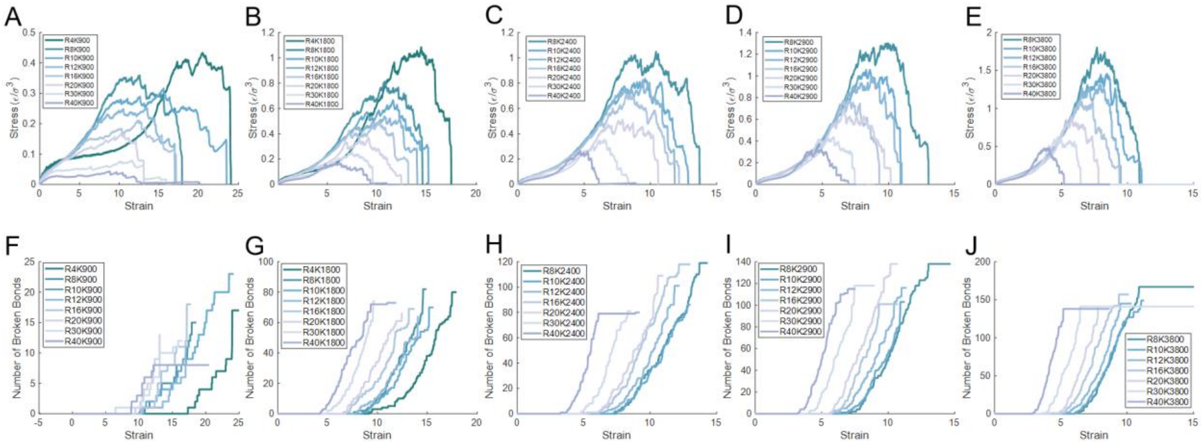

Figure S9. Stress-strain response and bond rupture behavior of networks with *N*=100 at different *K* and R. (A-E) Stress-strain curves for *K*=900, *K*=1800, *K*=2400, *K*=2900, and *K*=3800 respectively, with *R* ranging from 4 to 16. (F-J) Corresponding bond rupture profiles for the same systems, showing that bond breaking initiates earlier with increasing *R*, whereas larger *K* increases the total number of broken bonds at failure.

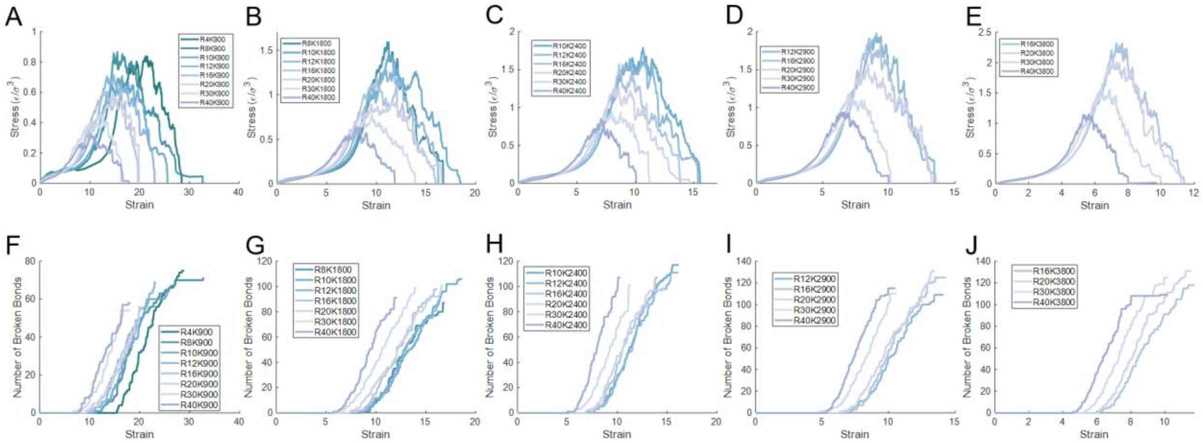

Figure S10. Stress-strain response and bond rupture behavior of networks with N=200 at different K and R. (A-E) Stress-strain curves for K=900, K=1800, K=2400, K=2900, and K=3800 respectively, with R ranging from 4 to 40. (F-J) Corresponding bond rupture profiles for the same systems, showing that bond breaking initiates earlier with increasing R, whereas larger K increases the total number of broken bonds at failure.

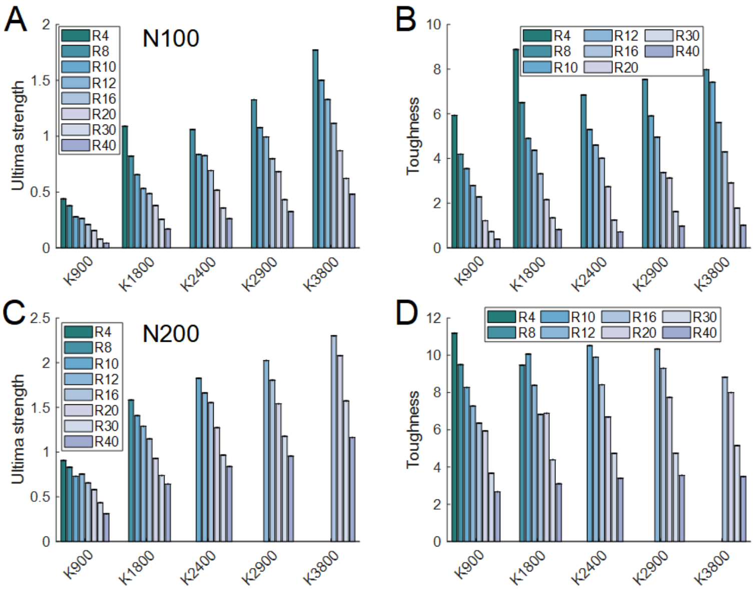

Figure S11. Mechanical response of SR networks with N=100 and N=200 at different R. (A) Ultimate strength and (B) toughness as a function of K for different R with N=100. (C) Ultimate strength and (D) toughness as a function of K for different R with N=200. Both properties decrease with increasing R. The values are averages over three independent simulations, and the minor error bars indicate standard deviations.

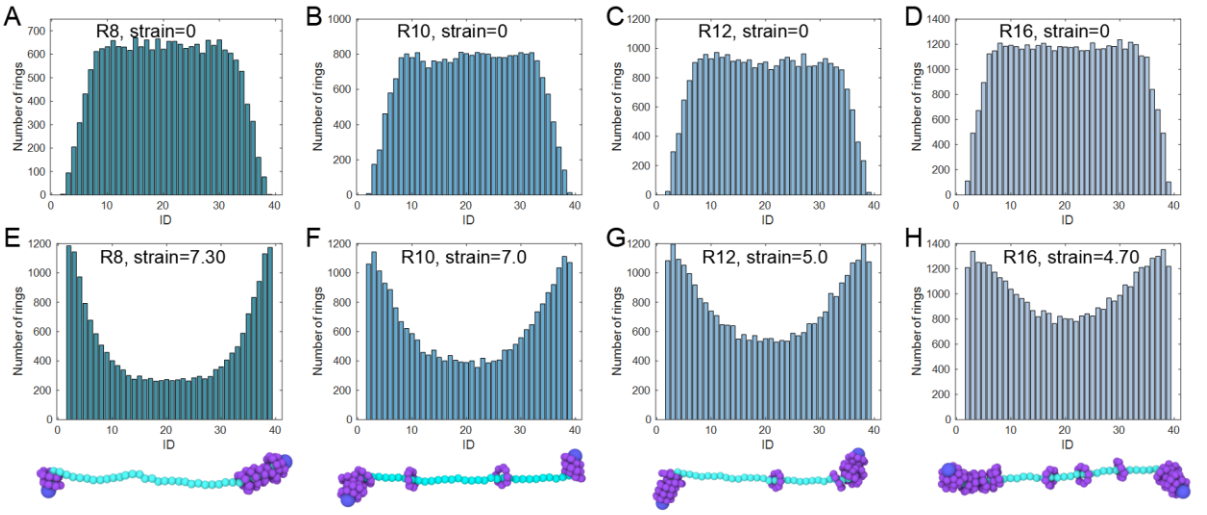

Figure S12. Distribution of rings along the polymer axial chains during stretching. (A)-(D) Distribution of ring centers of mass at the undeformed (strain=0) and the deformed state. Rings are initially distributed throughout the axial chains but gradually migrate toward the chain ends, transforming from an inverted "U-shaped" to a "U-shaped" profile. The schematic snapshots below each panel illustrate representative chain conformations for different R, highlighting the aggregation of rings near the chain ends at large strains.

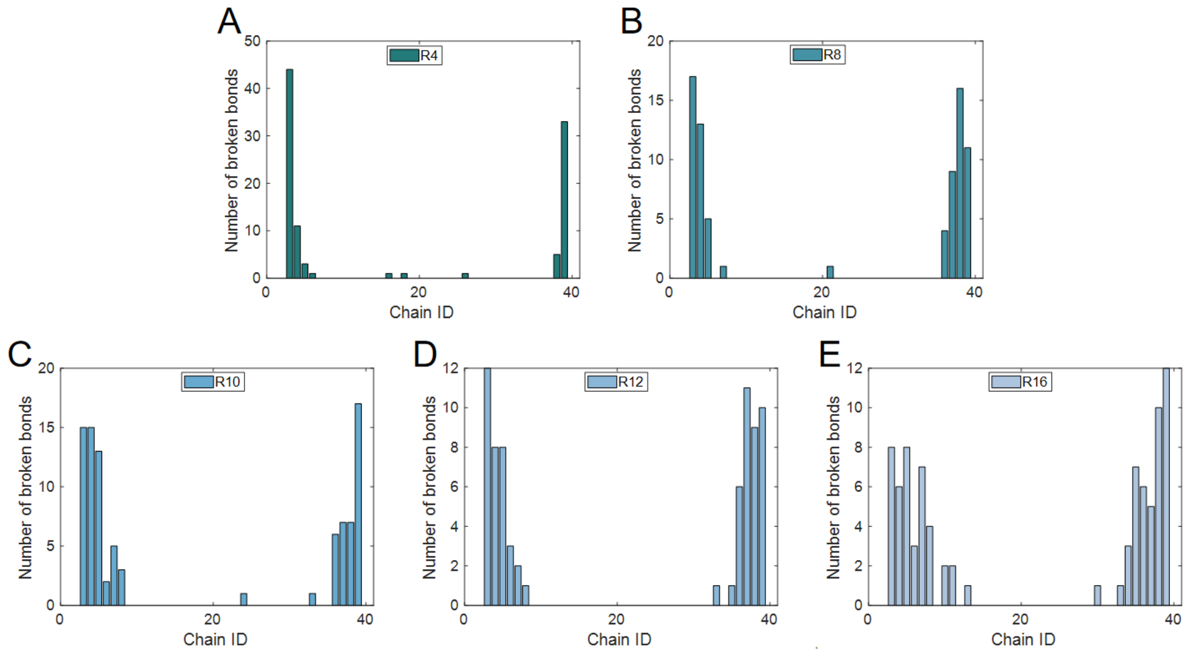

Figure S13. Distribution of bond rupture locations along the axial chains for networks with N=40 and K=3800 and different R: (A) R=4, (B) R=8, (C) R=10, (D) R=12, and (E) R=16. In all cases, broken

bonds are predominantly concentrated near the chain ends, whereas relatively few rupture events occur in the central region.

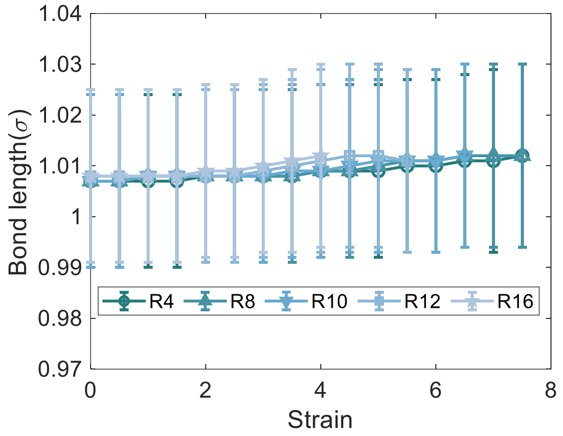

Figure S14. Average bond length as a function of strain for networks with R = 4, 8, 10, 12, and 16. The bond length remains nearly constant during deformation. Error bars represent standard deviations.

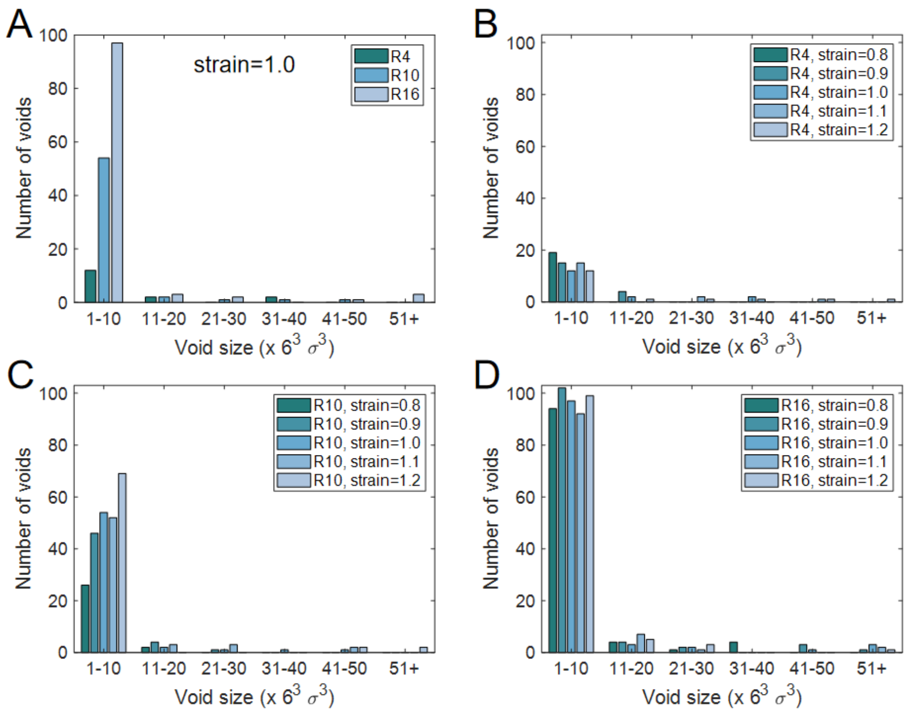

Figure S15. Void size distributions for N=40 and K=3800. (A) Void size distributions for systems with different ring numbers (R4, R10, and R16) at a strain of 1.0. (B-D) Void size distributions for systems with R4 (B), R10 (C), and R16 (D) at three neighboring strain values (0.8-1.2). Only minor

differences were observed among these distributions for each system, and the overall trends remained unchanged.

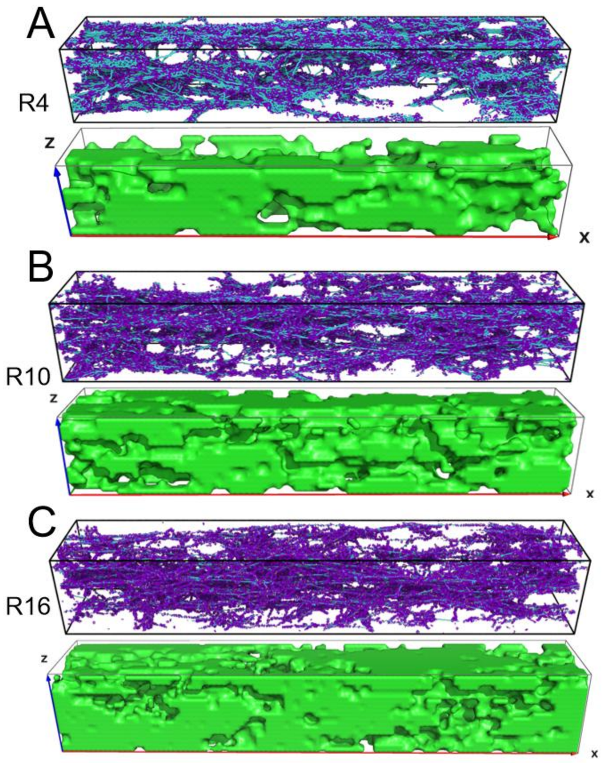

Figure S16. Representative polymer configurations and corresponding void structures at strain=5 for (A) R4, (B) R10 and (C) R16 of N40K3800 systems. For each system, the upper panel shows the polymer network configuration, and the lower panel shows the corresponding void morphology. At this large strain, voids percolate throughout the simulation box. Nevertheless, differences among network structures remain observable, with the R4 system exhibiting the smallest overall void compared to higher-R systems.

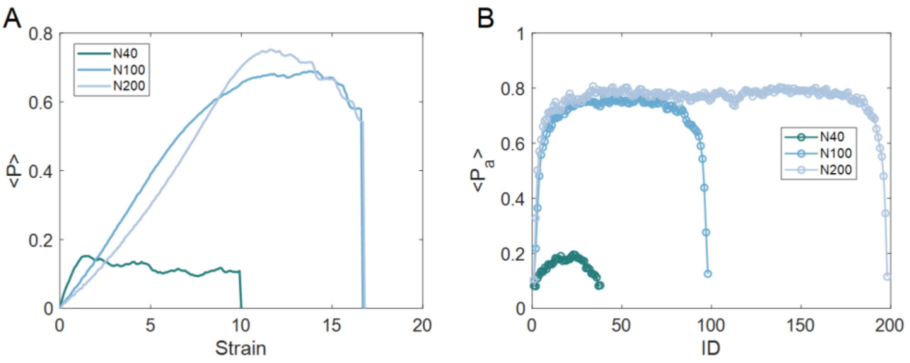

Figure S17. Chain orientation during deformation. (A) The average orientational order parameter $\langle P \rangle$ for networks with *N*=40, 100, and 200 at *R*=8 and *K*=1800. The results shows higher maximum alignment at longer axial chain length (*N*200). (B) Atomic order parameter $\langle P_a \rangle$ as a function of bead index, averaged over chains.

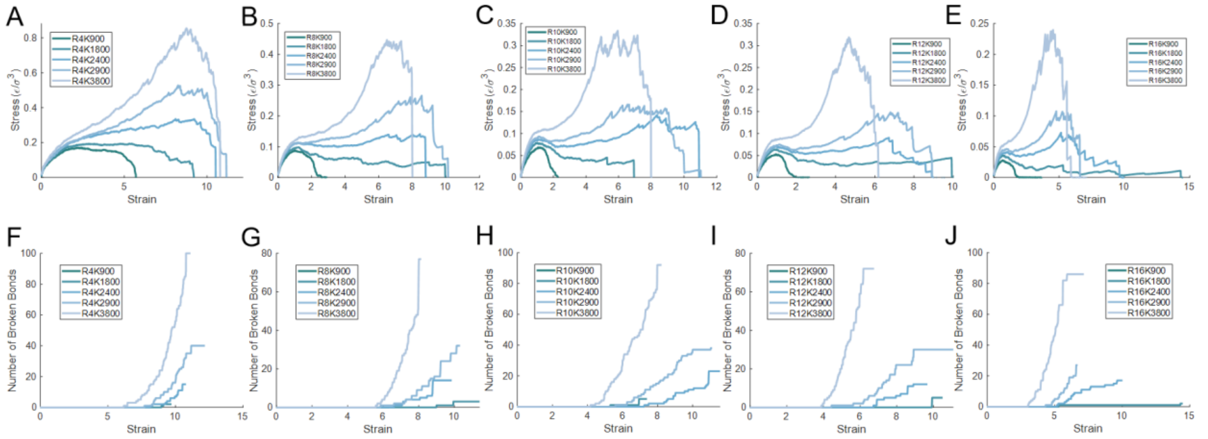

Figure S18. Stress-strain response and bond rupture behavior of networks with *N*=40 at different *K* and *R*. (A-E) Stress-strain curves for *R*=4, 8, 10, 12, and 16, respectively. (F-J) Corresponding bond rupture profiles for the same systems, showing that bond breaking initiates earlier with increasing *K*.

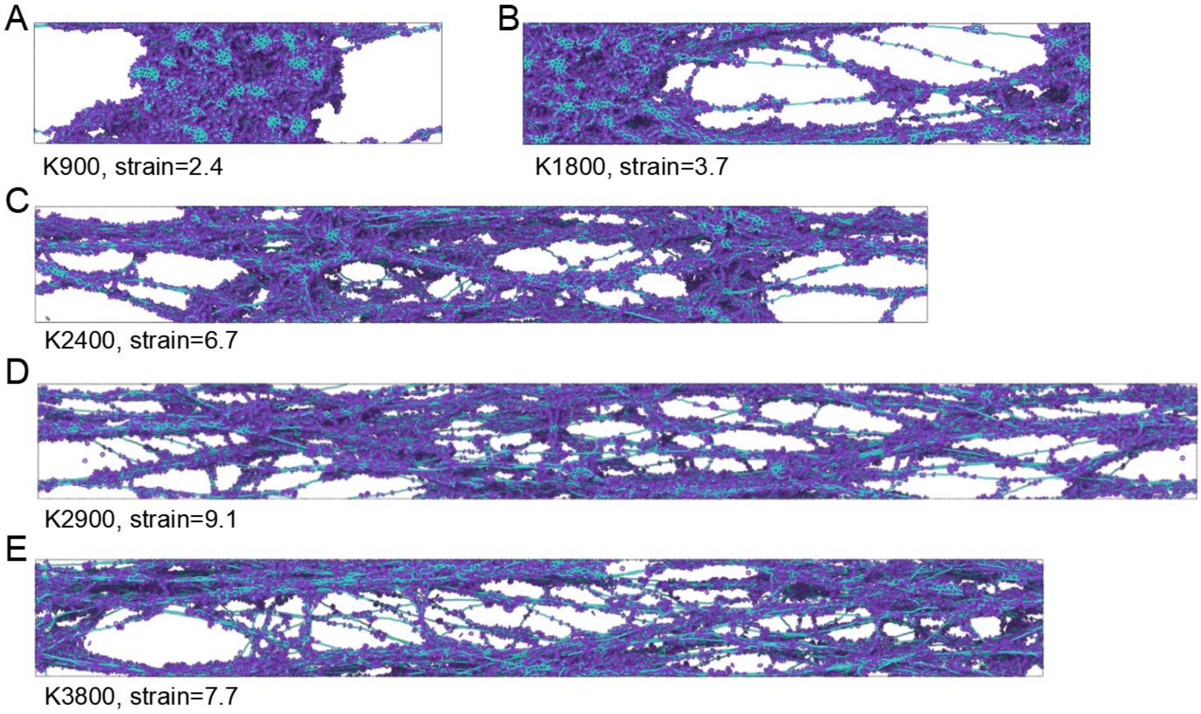

Figure S19. Representative network structures with N=40 and R=8 at large strains for different K. (A) K900, strain = 2.4; (B) K1800, strain = 3.7; (C) K2400, strain = 6.7; (D) K2900, strain = 9.1; (E) K3800, strain = 7.7. At small K, large voids form and the networks fail progressively even at small strains. By contrast, highly crosslinked systems deform more uniformly, with more homogeneous structural evolution.

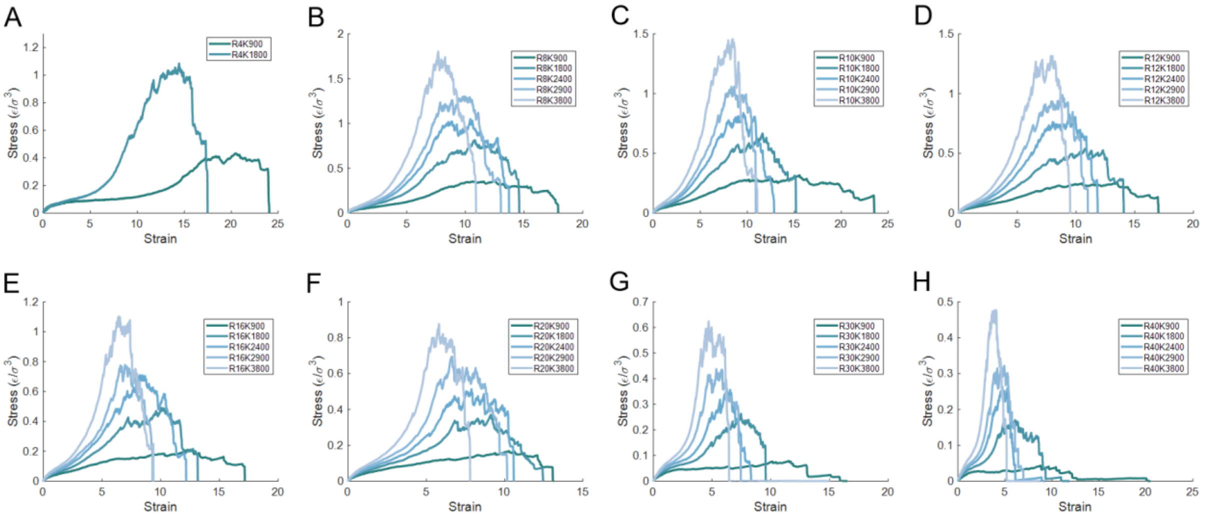

Figure S20. Stress-strain response of networks with N=100 at different K and R. (A-H) Stress-strain curves for R=4 to 40, respectively, with K=900 to 3800. For N=100 with R=4, the maximum possible K is 4 x 1000/2=2000, since the system contains 1000 chains. As a result, (A) shows only two curves.

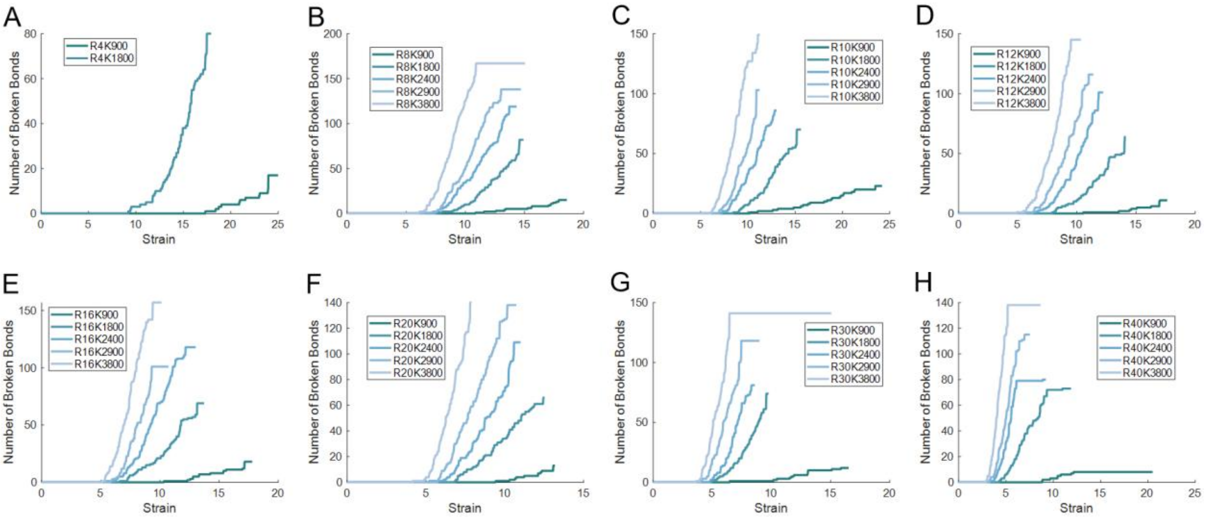

Figure S21. Number of broken bonds with N=100 at different K and R. (A-H) R=4 to 40, respectively, with K=900 to 3800. It shows that bond breaking initiates earlier with increasing K, whereas larger K increases the total number of broken bonds at failure. For N=100 with R=4, the maximum K is 4 x 1000/2=2000, since the system contains 1000 chains. As a result, (A) shows only two curves.

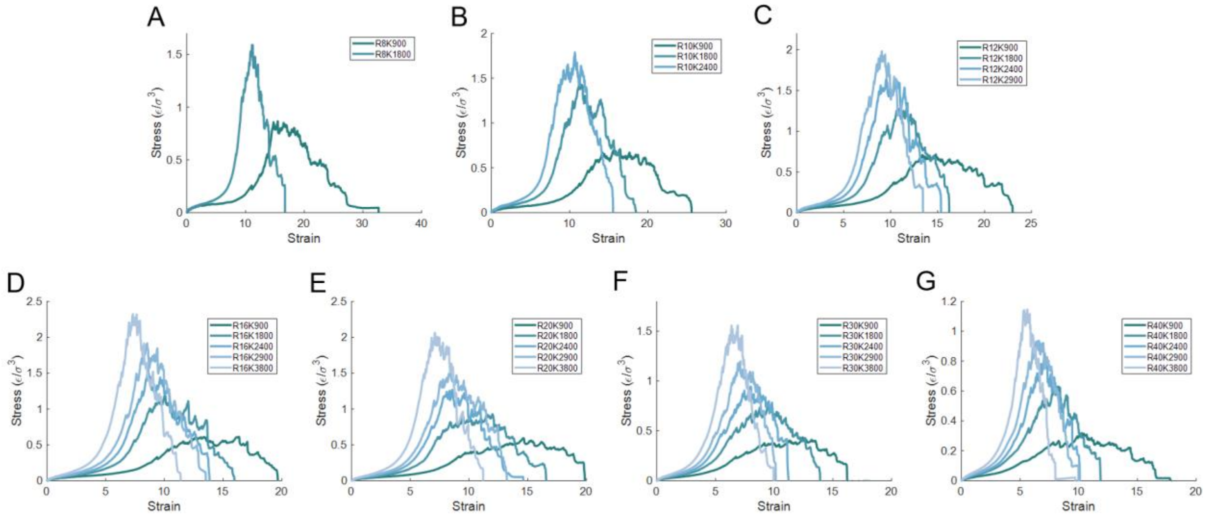

Figure S22. Stress-strain response of networks with N=200 at different K and R. (A-H) Stress-strain curves for R=4 to 40, respectively, with K=900 to 3800. For N=200 with R=4, the system has only 500 chains, and the maximum K is 4 x 500/2=1000. Therefore, for R=4, only a single curve is obtained and is not shown. As R increases, K also increases.

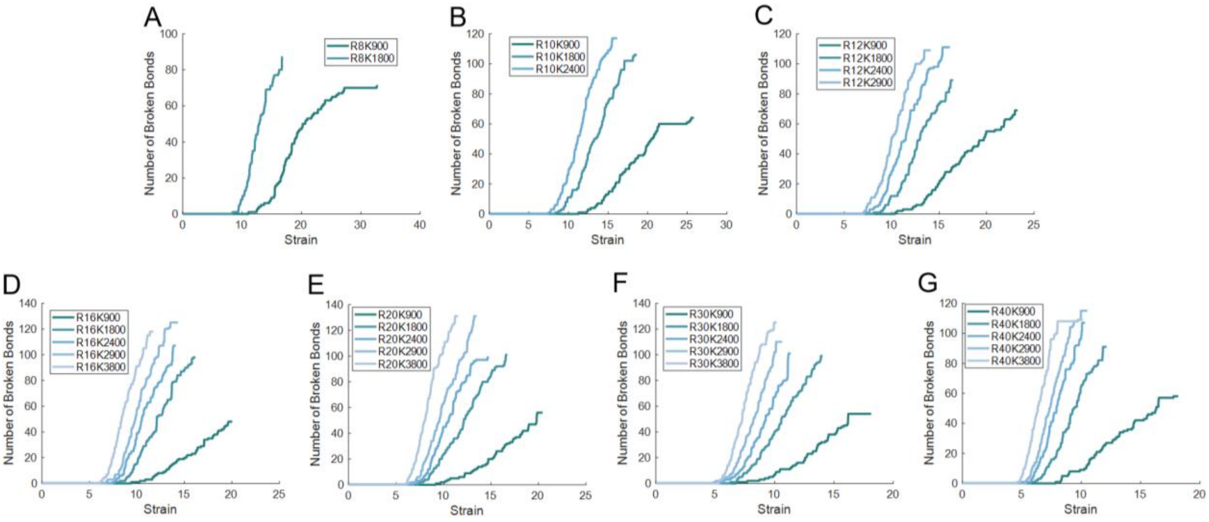

Figure S23. (A-H) Number of broken bonds with *N*=200 at different *K* and *R*. It shows that bond breaking initiates earlier with increasing *K*, whereas larger *K* increases the total number of broken bonds at failure. For *N*=200 with *R*=4, the system has only 500 chains, and the maximum *K* is 4 x 500/2=1000. Therefore, for *R*=4, only a single curve is obtained and is not shown. As *R* increases, *K* also increases.

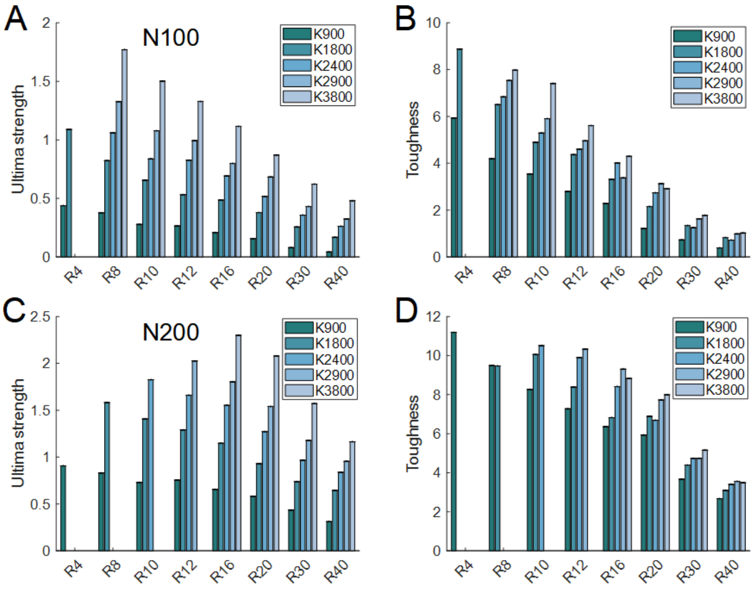

Figure S24. Mechanical response of SR networks with *N*=100 and *N*=200 at different *K* and *R*. (A) Ultimate strength and (B) toughness as a function of *R* for different *K* with *N*=100. (C) Ultimate strength and (D) toughness as a function of *R* for different *K* with *N*=200. Both properties increase with increasing *K*. The values are averages over three independent simulations, and the minor error bars indicate standard deviations.

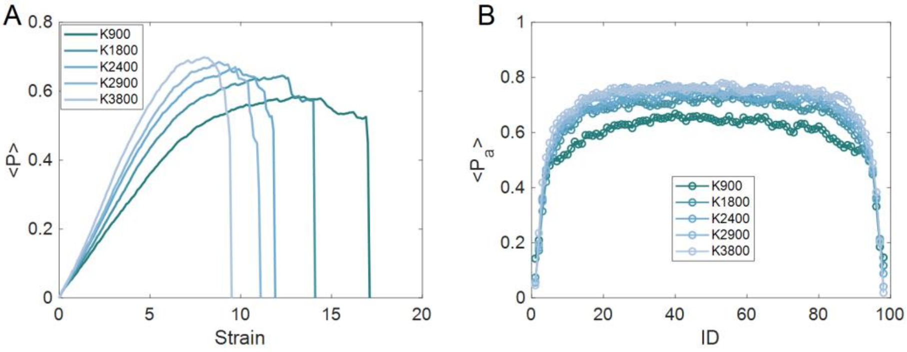

Figure S25. Chain orientation during deformation. (A) The average orientational order parameter ⟨*P*⟩ for networks with *K*=900, 1800, 2400, 2900, and 3800 at *R*=12 and *N*=100. The results shows higher

maximum alignment at larger K. (B) Atomic order parameter $\langle P_a \rangle$ as a function of bead index, averaged over chains.

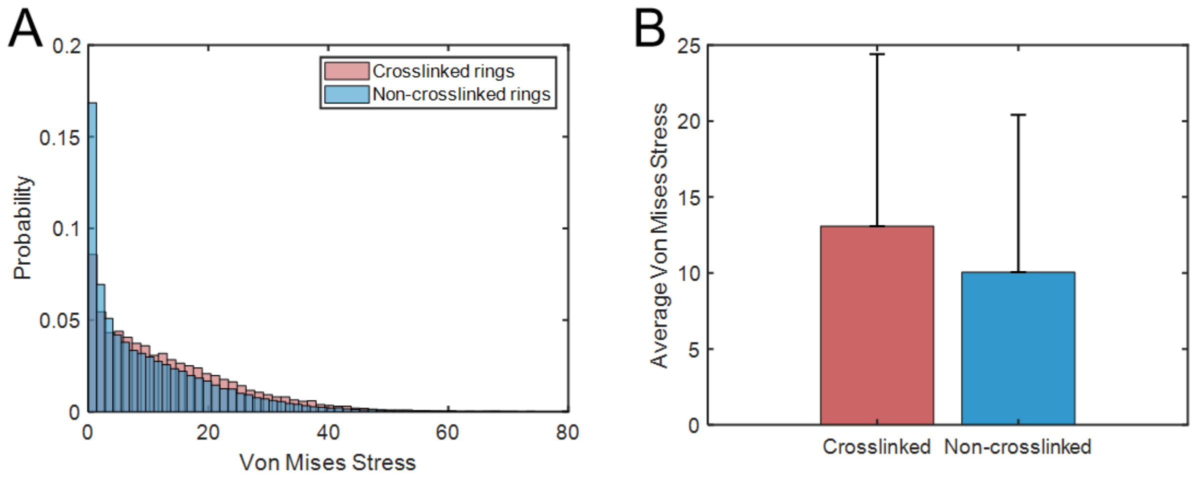

Figure S26. Local stress distribution and concentration in crosslinked and non-crosslinked rings for the N100R12K900 system at strain=7.5. (A) Probability distributions of von Mises stress of crosslinked and non-crosslinked rings. (B) Average von Mises stress for crosslinked and non-crosslinked rings, showing that crosslinked rings bear a higher load. Error bars represent standard deviations.

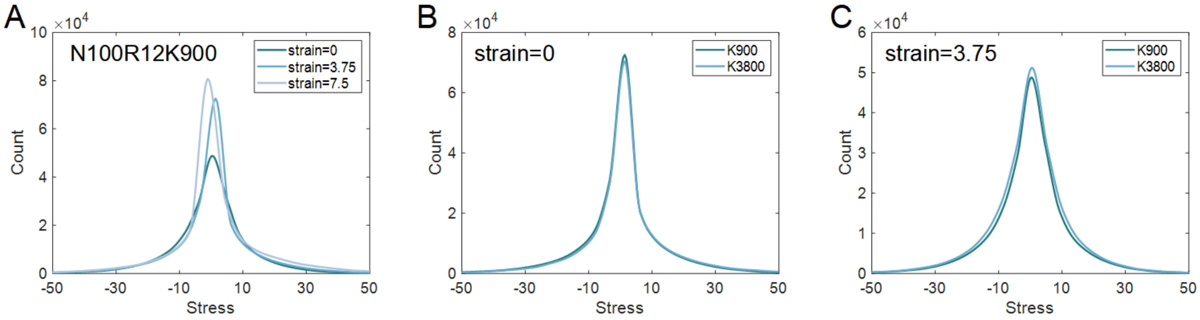

Figure S27. Local stress distribution along the stretching direction for SR networks with N=100, R=12, and different K. (A) Stress distribution for the K=900 system at strains of 0, 3.75, and 7.5. (B) and (C) Stress distribution between K=900 and K=3800 networks at strain = 0, and 3.75, respectively.

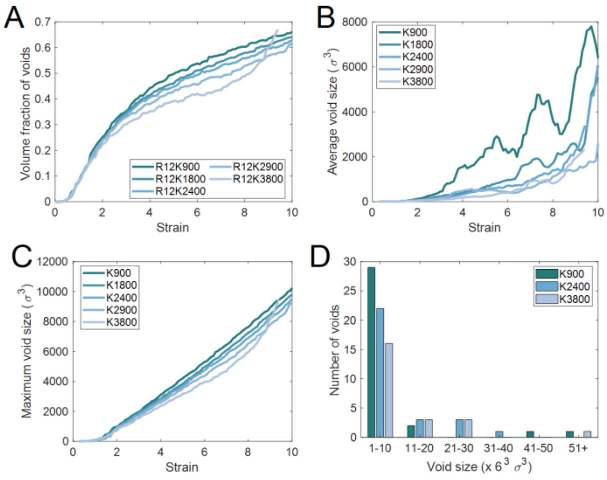

Figure S28. Quantification of void evolution for SR networks with N=100, R=12, and K=900-3800. (A) Total void volume fraction as a function of strain, showing a reduction with increasing K. (B) Average void size, (C) maximum void size and (D) void size distribution at strain=1 for networks with different K.

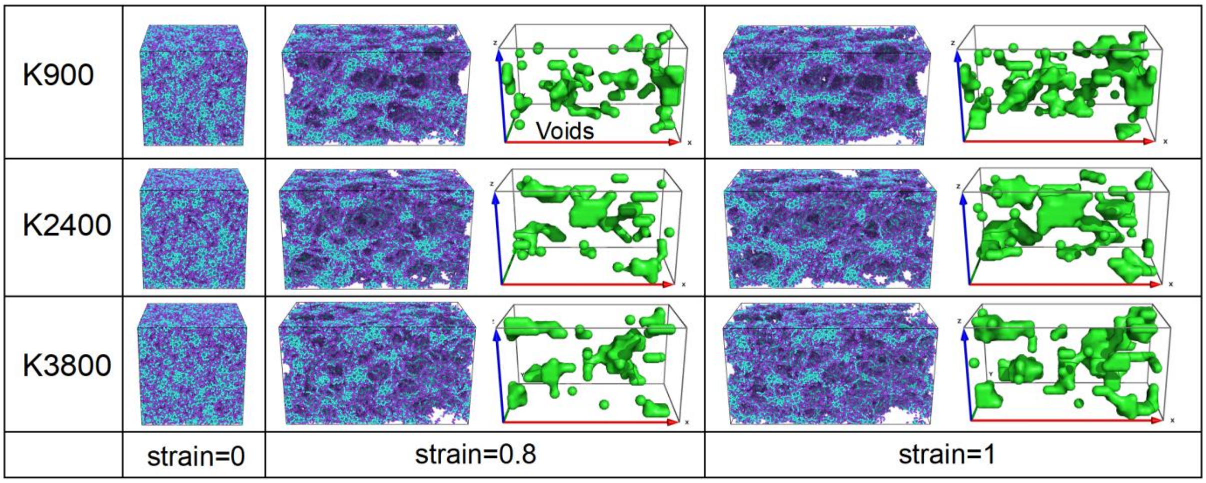

Figure S29. Representative snapshots of void evolution during deformation for networks with N=100, R=12 and K=900, 2400 and 3800. Snapshots are shown at strains of 0, 0.8, and 1. Green regions denote voids. Fewer and smaller voids are observed for K=3800. Beads are colored according to type: end beads (blue), axial beads (cyan), and ring beads (purple).

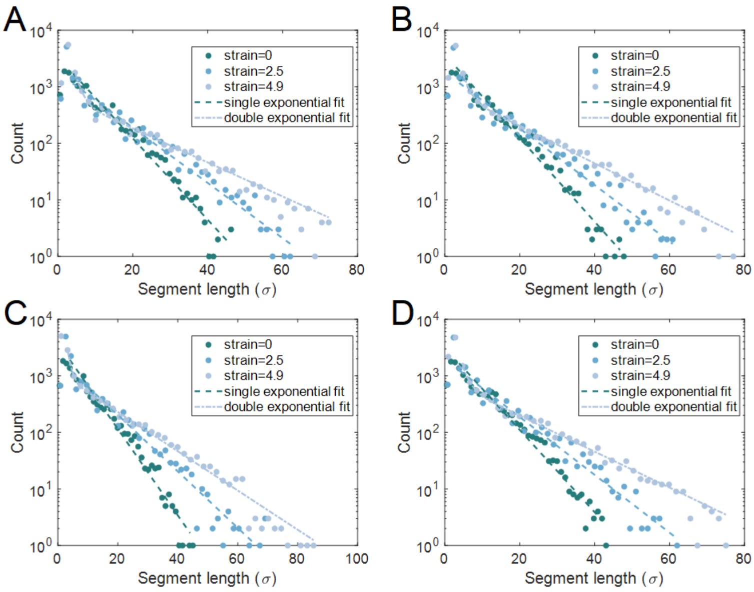

Figure S30. Segment length distributions at different strains for networks with varying K: (A) K = 1800, (B) K = 2400, (C) K = 2900, and (D) K = 3800. At strain = 0, all systems follow a single-exponential distribution, consistent with random ring placement along the backbone. With increasing strain, the distributions evolve into a double-exponential form, reflecting the emergence of both short and long segments due to ring sliding.

Reference

(1) Kremer, K.; Grest, G. S. Dynamics of Entangled Linear Polymer Melts: A Molecular-Dynamics Simulation. *J. Chem. Phys.* **1990**, *92* (8), 5057–5086. https://doi.org/10.1063/1.458541.

(2) Thompson, A. P.; Aktulga, H. M.; Berger, R.; Bolintineanu, D. S.; Brown, W. M.; Crozier, P. S.; In ’T Veld, P. J.; Kohlmeyer, A.; Moore, S. G.; Nguyen, T. D.; Shan, R.; Stevens, M. J.; Tranchida, J.; Trott, C.; Plimpton, S. J. LAMMPS - a Flexible Simulation Tool for Particle-Based Materials Modeling at the Atomic, Meso, and Continuum Scales. *Comput. Phys. Commun.* **2022**, *271*, 108171. https://doi.org/10.1016/j.cpc.2021.108171.

(3) Zhang, S.; Cao, Z.; Gu, X.; Ge, T. Polymer Thin Film Necking: Ductility from Entanglements and Plane Stress Condition. *Macromolecules* **2024**, *57* (13), 6221–6232. https://doi.org/10.1021/acs.macromol.4c00656.

(4) Xian, W.; Maiti, A.; Saab, A. P.; Li, Y. Understanding Viscoelasticity of an Entangled Silicone Copolymer via Coarse-Grained Molecular Dynamics Simulations. *Macromolecules* **2025**, acs.macromol.5c01192. https://doi.org/10.1021/acs.macromol.5c01192.

(5) Zhang, Z.; Hou, G.; Shen, J.; Liu, J.; Gao, Y.; Zhao, X.; Zhang, L. Designing the Slide-Ring Polymer Network with Both Good Mechanical and Damping Properties via Molecular Dynamics Simulation. *Polymers* **2018**, *10* (9), 964. https://doi.org/10.3390/polym10090964.
